# Metabolic profiling identifies *Qrich2* as a novel glutamine sensor that regulates microtubule glutamylation and mitochondrial function in mouse sperm

**DOI:** 10.1007/s00018-024-05177-4

**Published:** 2024-04-10

**Authors:** Guohui Zhang, Juncen Guo, Haoxuan Yang, Qing Li, Fei Ye, Yuelin Song, Dongsheng Xiong, Jiuzhi Zeng, Weiwei Zhi, Shuiqiao Yuan, Yunyun Lv, Tongtong Li, Yan Wang, Lu Liao, Dong Deng, Weixin Liu, Wenming Xu

**Affiliations:** 1 Department of Obstetrics and Gynecology, Joint Laboratory of Reproductive Medicine (SCU-CUHK), Key Laboratory of Obstetric, Gynecologic and Pediatric Diseases and Birth Defects of Ministry of Education, West China Second University Hospital, Sichuan University, Chengdu, 610041 China; 2 Key Laboratory of Reproductive Medicine, Sichuan Provincial Maternity and Child Health Care Hospital, Chengdu, 610000 China; 3 Institute Reproductive Health, Tongji Medical College, Huazhong University of Science and Technology, Wuhan, 430030 China; 4 Reproduction Medical Center of West China Second University Hospital, Key Laboratory of Obstetric, Gynecologic and Pediatric Diseases and Birth Defects of Ministry of Education, Sichuan University, Chengdu, 610041 China; 5 Key Laboratory of Sichuan Province for Fishes Conservation and Utilization in the Upper Reaches of the Yangtze River, College of Life Sciences, Neijiang Normal University, Neijiang, 641100 China; 6 Puhua Bioscience, Chengdu, 610000 China

**Keywords:** *Qrich2*, Microtubule glutamylation, Gln/Glu metabolism, Sperm flagella, Mitochondrial function

## Abstract

In our prior investigation, we discerned loss-of-function variants within the gene encoding glutamine-rich protein 2 (QRICH2) in two consanguineous families, leading to various morphological abnormalities in sperm flagella and male infertility. The *Qrich2* knockout (KO) in mice also exhibits multiple morphological abnormalities of the flagella (MMAF) phenotype with a significantly decreased sperm motility. However, how ORICH2 regulates the formation of sperm flagella remains unclear. Abnormal glutamylation levels of tubulin cause dysplastic microtubules and flagella, eventually resulting in the decline of sperm motility and male infertility. In the current study, by further analyzing the *Qrich2* KO mouse sperm, we found a reduced glutamylation level and instability of tubulin in *Qrich2* KO mouse sperm flagella. In addition, we found that the amino acid metabolism was dysregulated in both testes and sperm, leading to the accumulated glutamine (Gln) and reduced glutamate (Glu) concentrations, and disorderly expressed genes responsible for Gln/Glu metabolism. Interestingly, mice fed with diets devoid of Gln/Glu phenocopied the *Qrich2* KO mice. Furthermore, we identified several mitochondrial marker proteins that could not be correctly localized in sperm flagella, which might be responsible for the reduced mitochondrial function contributing to the reduced sperm motility in *Qrich2* KO mice. Our study reveals a crucial role of a normal Gln/Glu metabolism in maintaining the structural stability of the microtubules in sperm flagella by regulating the glutamylation levels of the tubulin and identifies *Qrich2* as a possible novel Gln sensor that regulates microtubule glutamylation and mitochondrial function in mouse sperm.

**Supplementary Information:**

The online version contains supplementary material available at 10.1007/s00018-024-05177-4.

## Introduction

Approximately 15% of couples at childbearing age suffer fertility problems owing to environmental changes and life stress, of which male infertility accounts for about 50% [24]. Sperm abnormalities primarily manifest as asthenospermia, oligozoospermia, azoospermia, and teratozoospermia, collectively constituting the leading causes of male infertility [15]. Asthenospermia is usually caused by abnormal structure and/or function of flagella, which play a critical role in the reproductive process, such as capacitation and fertilization [28]. Furthermore, serving as the axial structure of sperm flagella, the axoneme is composed of tubulin and is arranged as a typical “9 + 2” structure, including nine pairs of peripheral microtubules and one pair of central microtubules. Microtubules are structures comprised of highly conserved α-TUBULIN and β-TUBULIN, possessing extensive functions in maintaining the morphology and motility of cilia and flagella [13]. Various factors, such as the interaction with microtubule-associated proteins, the differential expression of the gene coding tubulin isoforms, and the post-translational modifications (PTMs) of tubulin are coordinatively involved in determining the diversity of microtubule functions. PTMs of tubulin, which have been recently designed as tubulin codes, have emerged as the central player involved in many biological processes, including spermatogenesis and sperm functional regulation [1, 3, 4, 7, 19, 35].

Several PTMs types in tubulin have been proven to regulate microtubule stability and sperm motility. For instance, the knockout (KO) of Alpha-tubulin N-acetyltransferase 1 (ATAT1) in mice induces increased acetylation levels of tubulin and decreased stability of microtubules in sperm flagella, thereby reducing sperm motility and fertility [13]. In addition, the loss of function in tubulin deacetylase histone deacetylase 6 (HDAC6) causes increased acetylation of tubulin and decreased sperm motility [22]. These studies indicate the necessity of appropriate acetylation levels of tubulin to maintain the stability of microtubules and normal sperm motility. Interestingly, in the double KO mice of Tubulin tyrosine ligase like 3 and 8 (*Ttll3* and *Ttll8*), an anomalous swimming pattern of flagella with the circular movement is observed owing to the significant decrease in tubulin glycylation levels, even though the flagella are normally assembled [6]. Detailed investigation of the ultrastructure in flagella shows a disorderly arranged outer dynein arm and inner dynein arm in *Ttll3* and *Ttll8* double KO mice, which causes the reduced sperm motility and fertility of double KO mice [6]. Moreover, tubulin glutamylation homeostasis has recently been shown to be critical for regulating microtubule stability and sperm flagella development [8, 20, 27]. *Ttll5* KO decreases tubulin glutamylation levels and increases the loss of central pairs in the axoneme of flagella [16]. At the same time, the loss of function of cytosolic carboxypeptidase-like protein 5 (CCP5) causes accumulated tubulin with polyglutamylation, disorganized microtubules, and stacked organelles in sperm [9].

Our previous study identified loss-of-function mutations in the gene encoding glutamine-rich protein 2 (QRICH2) in patients from two consanguine families and found multiple morphological abnormalities of flagella (MMAF) in sperm of the patients [25]. However, the specific regulatory mechanism of QRICH2 involved in sperm tubulin formation has not been fully clarified. Notably, the core constituents of the typical “9 + 2” structure are composed of tubulin, and we further observed the missing and/or disordered arrangement of microtubule constituents in flagella of the patients carrying *QRICH2* mutations; thus, we aims to understand whether the Glutamine (Gln) enriched protein QRICH2 is involved in regulating the microtubules stability and the development of flagella by affecting the PTMs of glutamylation. Thus, in this study, we utilized the conventional *Qrich2* KO mouse model to comprehensively investigate the function of QRICH2 in regulating the PTMs and the stability of tubulin in sperm flagella. Our data clarify the critical role of amino acid metabolism in spermatogenesis and suggest regulating Gln/ Glutamate (Glu) metabolism as an effective strategy for patients with decreased sperm microtubule glutamylation to improve their sperm motility and fertilizing capability during in vitro fertilization.

## Results

### QRICH2 is involved in regulating Gln/Glu metabolism and conversion

To further comprehensively reveal the function of QRICH2 in spermatogenesis, the functional domains of QRICH2 were analyzed. First, we constructed the phylogenetic tree of QRICH2 and annotated the conservative domains. As depicted in Fig. S1A, Homo sapiens' QRICH2 comprises three conserved domains: DUF4795, Glutenin_hmw, and SMC_prok_B. The DUF4795 domain exhibits a high degree of conservation across various species, suggesting its potential involvement in fundamental life processes (Fig. S1B). Interestingly, the Glutenin_hmw domain specifically appears in the QRICH2 of mammals (Fig. S1B), indicating that its emergence might be related to the characteristics of mammalian sperm function in the process of evolution. In addition, we found that QRICH2, particularly the Glutenin_hmw domain, was rich in Gln (Fig. S1C), suggesting the potential role of QRICH2 in regulating Gln metabolism, probably as a sensor or regulator.

**Fig. 1:**
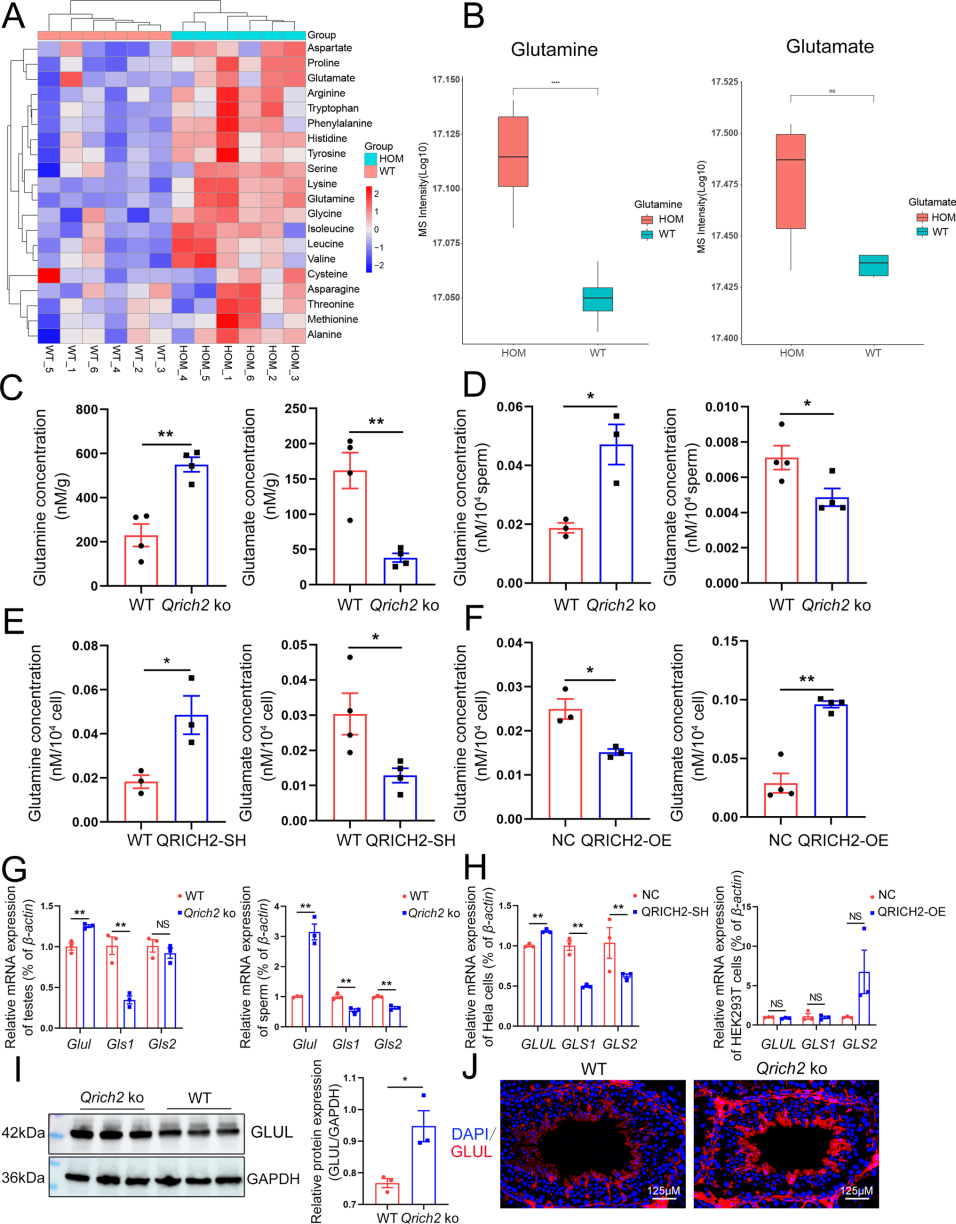
Disordered Gln/Glu metabolism/conversion mediates the decrease in tubulin glutamylation levels in sperm flagella of *Qrich2* KO mice. WT, wild-type; HOM or *Qrich2* KO, *Qrich2* knockout; NC, normal control; QRICH2-SH, QRICH2 knockdown; QRICH2-OE, QRICH2 overexpression. **A** Hierarchical cluster analysis of 20 amino acids in WT and *Qrich2* KO mice. N = 6, the depth of red or blue represents the levels of the concentration. **B** Reduced concentration of Gln was observed in the testes of *Qrich2* KO mice, while the concentration of Glu remained unchanged. N = 6, Student’s *t* test, ns, *P* > 0.05, ****P <  = 0.0001, error bars, s.e.m. **C**, **D** The increased Gln concentration and decreased Glu concentration were observed in the testes (**C**) and sperm (**D**) of *Qrich2* KO mice*.* N = 3 or N = 4, Student’s *t* test, *P < 0.05, **P < 0.01, error bars, s.e.m. **E**, **F** Knockdown of QRICH2 increased the Gln concentration and decreased the Glu concentration (**E**); On the contrary, overexpression of QRICH2 decreased the Gln concentration and increased the Glu concentration (**F**). N = 3 or N = 4; Student’s *t* test, *P < 0.05, **P < 0.01, error bars, s.e.m. **G** *Qrich2* KO caused disordered expression of genes related to Gln/Glu metabolism/conversion in mouse testes and sperm (N = 3, Student’s *t* test, **P < 0.01, NS, *P* > 0.05, error bars, s.e.m. **H** Knockdown of QRICH2 in HeLa cells induced disordered expression of genes related to Gln/Glu metabolism/conversion, while overexpression of QRICH2 showed no significant effect on the expression of these genes. N = 3, Student’s *t* test, NS, *P* > 0.05, **P < 0.01, error bars, s.e.m. **I**, **J** Western blot (**I**) and immunofluorescence (**J**) showed increased expression of GLUL in the testes of *Qrich2* KO mice. N = 3, Student’s *t* test, *P < 0.05, error bars, s.e.m. DAPI, blue; GLUL, red; scale bars, 125 µm

Thus, we conducted the targeted amino acid metabolomics analysis to further explore the role of QRICH2 in amino acid metabolism. The changes in amino acid concentrations showed high uniformity between wild-type (WT) and *Qrich2* KO groups (Fig. 1a). Compared with WT mouse testes, accumulation of major amino acids, including Gln, was observed in KO mouse testes, while the changes of Glu showed no significance (Fig. 1b). In addition, the pathway analysis showed that the differential metabolites were involved in amino acid metabolic pathways (Fig. S2A. B), and the enriched ontology clusters analysis of QRICH2’s interacting proteins also showed its involvement in amino acid metabolism (Fig. S2C). These results suggested the potential role of QRICH2 in regulating amino acid metabolism. Subsequently, we used a Gln and Glu concentration assay kit to verify the Gln and Glu concentrations in the testes and sperm in vitro. The results showed an increased Gln concentration and decreased Glu concentration in both testes and sperm of *Qrich2* KO mice compared with WT mice (Fig. 1c, d). At the cellular level, the knockdown of QRICH2 increased the concentration of Gln and reduced the concentration of Glu (Fig. 1e). In contrast, overexpression of QRICH2 reduced Gln concentration and increased Glu concentration (Fig. 1f). These results indicated that QRICH2 may play a crucial role in the mutual conversion of Gln and Glu.

To further elucidate the mechanisms by which QRICH2 regulates Gln/Glu metabolism/conversion, we next detected the expression of genes including glutamine synthase (*Glul*) and glutaminase (*Gls*), which are responsible for the mutual conversion of Gln/Glu. There was an increased expression of *Glul* and a decreased expression of *Gls1* in the *Qrich2* KO sperm and testes (Fig. 1g). Interestingly, the expression levels of *Gls2* were reduced in *Qrich2* KO sperm, not testes (Fig. 1g). Moreover, we further detected the expression of these genes in cells with QRICH2 knockdown or overexpression. The knockdown of QRICH2 increased the expression of *GLUL* and reduced the expression of *GLS1* and *GLS2* (Fig. 1h). In contrast, the overexpression of QRICH2 showed no significant effect on the expression of these genes (Fig. 1h). Moreover, western blot and immunofluorescence analyses verified that the protein levels of GLUL in testes of *Qrich2* KO mouse were significantly increased, consistent with its mRNA levels (Fig. 1i, j). Together, our data indicate that QRICH2 plays a crucial role in regulating Gln/Glu metabolism and conversion, thereby affecting the concentration of Gln and Glu.

### QRICH2 depletion reduces glutamylation levels and expression of α-TUBULIN in sperm flagella

**Fig. 2:**
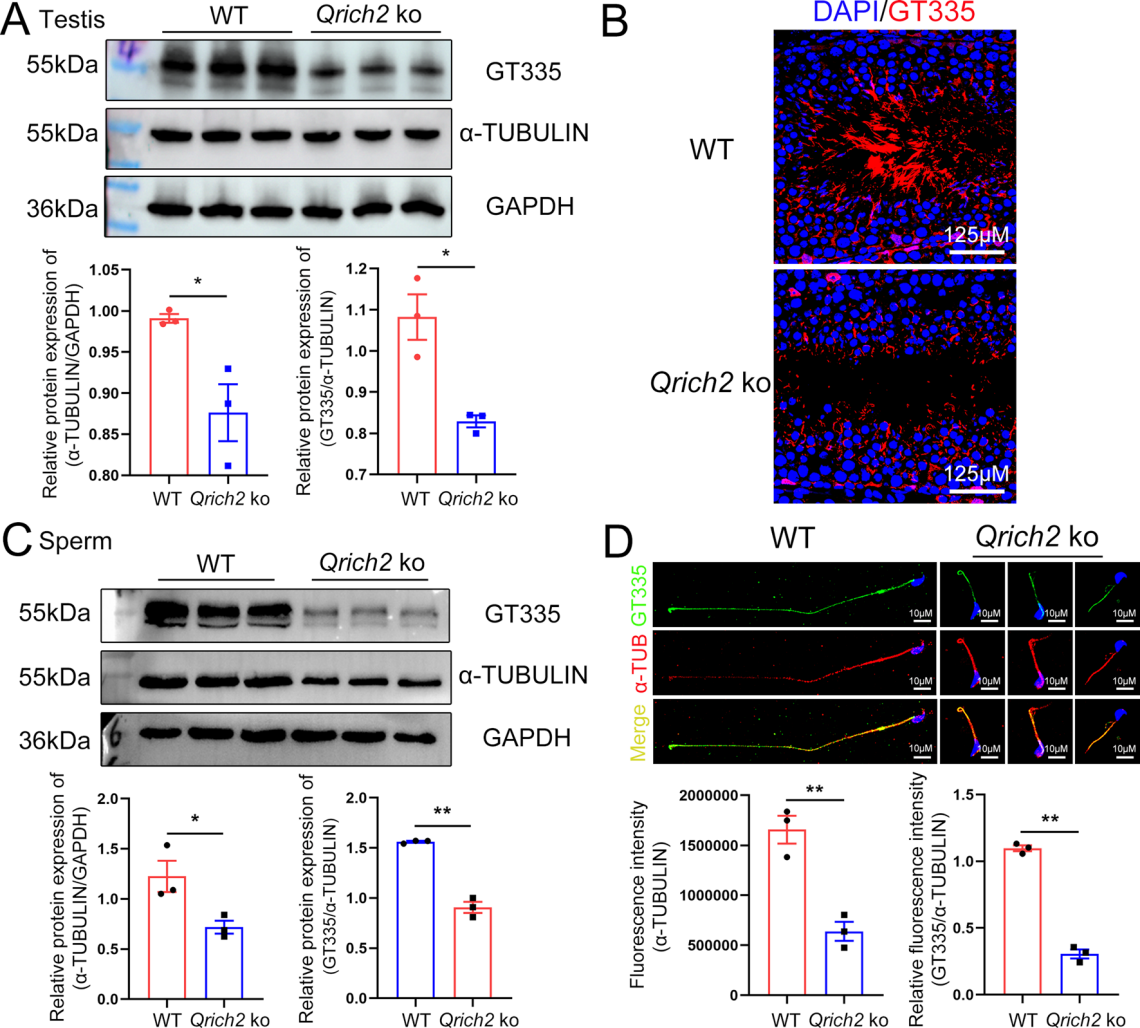
QRICH2 positively regulates the glutamylation levels of tubulin in flagella of mouse sperm. WT, wild-type; *Qrich2* KO, *Qrich2* knockout. (**A**, **B**) The western blot (**A**) and immunofluorescence (**B**) showed reduced expression of α-TUBULIN and decreased tubulin glutamylation levels in the testes of *Qrich2* KO mice. The grayscale analysis of protein bands was shown in the bottom panel of **A**. N = 3, Student’s *t* test, *P < 0.05, error bars, s.e.m. DAPI, blue; GT335, red; scale bars, 125 µm. **C**, **D** The reduced expression of α-TUBULIN and decreased tubulin glutamylation levels in sperm of *Qrich2* KO mice were observed by western blot (**C**) and immunofluorescence (**D**). The grayscale and fluorescence intensity analysis were shown in the bottom panel. N = 3, Student’s *t* test, *P < 0.05, **P < 0.01, error bars, s.e.m. DAPI, blue; GT335, green; α-TUB, α-TUBULIN, red; scale bars, 10 µm

Appropriate concentrations of Gln and Glu are necessary for normal tubulin glutamylation levels because Glu acts as the substrate for tubulin glutamylation and can be converted from Gln [27], while microtubules with low tubulin glutamylation levels are unstable and easier to depolymerize [27]. To confirm whether the disrupted Gln/Glu metabolism resulting from *Qrich2* KO further induces disregulated microtubule glutamylation levels, we detected the glutamylation levels of tubulin in *Qrich2* KO mouse. The glutamylation levels (indicated by GT335) and the expression of α-TUBULIN were significantly reduced in testes and sperm of *Qrich2* KO mouse (Fig. 2a–d). Subsequently, we further investigated the regulation effects of QRICH2 at the cellular level in vitro. The overexpression of QRICH2 in HeLa cells increased the glutamylation levels of tubulin (Fig. S3A). However, what is the mechanism for the decrease in tubulin glutamylation levels in sperm of *Qrich2* KO mice? We detected the expression of genes involved in tubulin glutamylation regulation, including ATP/GTP binding protein-like 5 (*Abgl5*), which reduces the glutamylation, and *Ttll4,* which promotes the glutamylation of tubulin. Increased expression of *Agbl5* and reduced expression of *Ttll4* in testes and sperm of *Qrich2* KO mice were observed (Fig. S3B). Moreover, knockdown of QRICH2 increased the expression of *AGBL5* and reduced the expression of *TTLL1* and *TTLL4* (Fig. S3C, left panel), whereas the overexpression of QRICH2 showed no significant effect on the expression of these genes (Fig. S3C, right panel). The variability in gene response to overexpression of QRICH2 might be attributed to the selected cell lines, as these genes may not necessarily exhibit a robust response to QRICH2 overexpression in HEK293 cells. From the current results, the disordered gene expression induced decrease in Glu concentration and the disordered expression of molecules involved in regulating tubulin glutamylation levels together mediated the decrease of tubulin glutamylation levels in *Qrich2* KO mice.

### QRICH2 interacts with α-TUBULIN to maintain the stability of microtubules

**Fig. 3:**
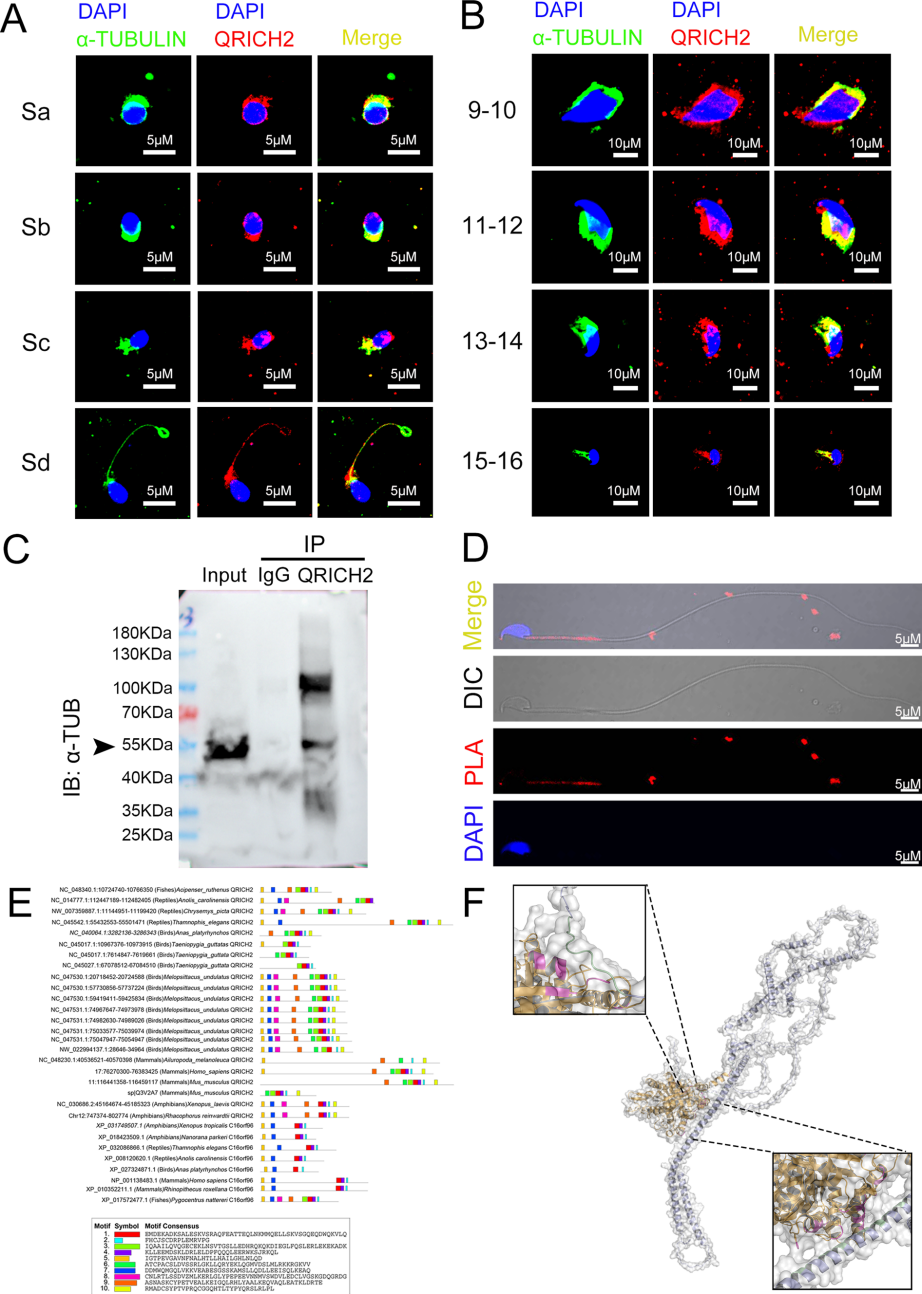
QRICH2 maintains the glutamylation levels and stability of tubulin through interaction with tubulin. **A**, **B** Co-localization of QRICH2 and α-TUBULIN was observed in spermatogenic cells with different developmental stages of humans (**A**) and mice (**B**). DAPI, blue; α-TUBULIN, green; QRICH2, red; scale bars, 5 µm (**A**), 10 µm (**B**); Sa-d and 9–16 represent spermatogenic cells of different developmental stages in humans and mice. **C**, **D** Co-IP (**C**) and Duolink PLA (**D**) experiments indicated the interaction between QRICH2 and α-TUBULIN. **E** Motifs identified in QRICH2 across several species. **F** Structural model of the QRICH2/α-TUBULIN complex. Two regions on QRICH2 (in magenta helix including His107, Ser110, Glu114, Gly117, Asp118, Glu120, Lys121, Ile124, Thr125, Asn128, Leu129, Asp132, Lys136, Asp139, Leu143, Tyr144, Gly146, Ile147, Leu150, Asp151, Lys154-157Arg and loop including His570, Asp572, Pro575-Arg584) and the corresponding binding sites on α-TUBULIN (in green) were highlighted in opaque New Cartoon representations and indicated by close-up views. The other parts of QRICH2/α-TUBULIN were represented in transparent orange and ice-blue

A previous study indicated the role of tubulin glutamylation levels in maintaining microtubule stability, the decrease in tubulin glutamylation levels caused instability and easier degradation of microtubules [27]. In previous results, we demonstrated that QRICH2 promoted the glutamylation of tubulin and maintained the stability of microtubules. We next interrogated how did this happen? By performing immunofluorescence staining, co-localization of QRICH2 and α-TUBULIN was observed in elongating spermatids (at stage critical for flagella assembly and extension, Fig. 3a, b) (12). During 9–12 developmental stages of elongating spermatids, QRICH2 was observed to be located in the transient structure of the manchette (Fig. 3a, b), which was composed of tubulin and played a vital role in spermatogenesis [17]. Subsequently, co-immunoprecipitation (Co-IP) and Duolink PLA experiments were conducted and indicated the interaction between QRICH2 and α-TUBULIN (Fig. 3c, d). Interestingly, the interaction between QRICH2 and α-TUBULIN was mainly concentrated in the middle piece of the flagella (Fig. 3d). To further map the binding site of α-TUBULIN on QRICH2, the sequence and structural characteristics of QRICH2 were investigated. First, we explored the conserved motifs of QRICH2 using the MEME online tool across species, and a total of 10 motifs were identified (Fig. 3e). In parallel, structural models of QRICH2 and α-TUBULIN were constructed by homology modeling, and then we employed protein–protein flexible docking technology to assess intermolecular interactions between them. Two regions on QRICH2 (in magenta helix ranging from His107 to Arg157 and loop ranging from His570 to Arg584)—including His107, Ser110, Glu114, Gly117, Asp118, Glu120, Lys121, Ile124, Thr125, Asn128, Leu129, Asp132, Lys136, Asp139, Leu143, Tyr144, Gly146, Ile147, Leu150, Asp151, Lys154-157Arg, His570, Asp572, and Pro575-Arg584—were predicted to interact with α-TUBULIN through intermolecular hydrogen bonds and hydrophobic interactions (Fig. 3f). Interestingly, the predicted interacting regions on QRICH2 almost all overlapped with the conserved 3rd motif Val108-Arg157, except for His570, Asp572, and Pro575-Arg584 (Fig. 3e, f). In addition, the reduced glutamylation levels and expression of α-TUBULIN in sperm of *Qrich2* KO mice were partially rescued by incubation of QRICH2 N-terminal purified protein (Fig. S3D), and meanwhile, the N-terminal purified protein was able to promote polymerization of α-TUBULIN in vitro (Fig. S3E). Moreover, the increased α-TUBULIN ubiquitination levels were observed in the testes of *Qrich2* KO mice and cells with QRICH2 knockdown (Fig. S3F). According to these findings, we inferred that the increased ubiquitination levels of α-TUBULIN were involved in medicating the reduced expression and stability of α-TUBULIN in the sperm of *Qrich2* KO mice. Furthermore, the interaction between QRICH2 and α-TUBULIN could play an important role in ensuring the stability and normal function of tubulin. It can be inferred that QRICH2 promoted the glutamylation of tubulin and thus increased the stability of tubulin during flagellar development.

### Decreased sperm motility and abnormal microtubule structure of flagella are observed in mice with absent Gln/Glu in diets

**Fig. 4:**
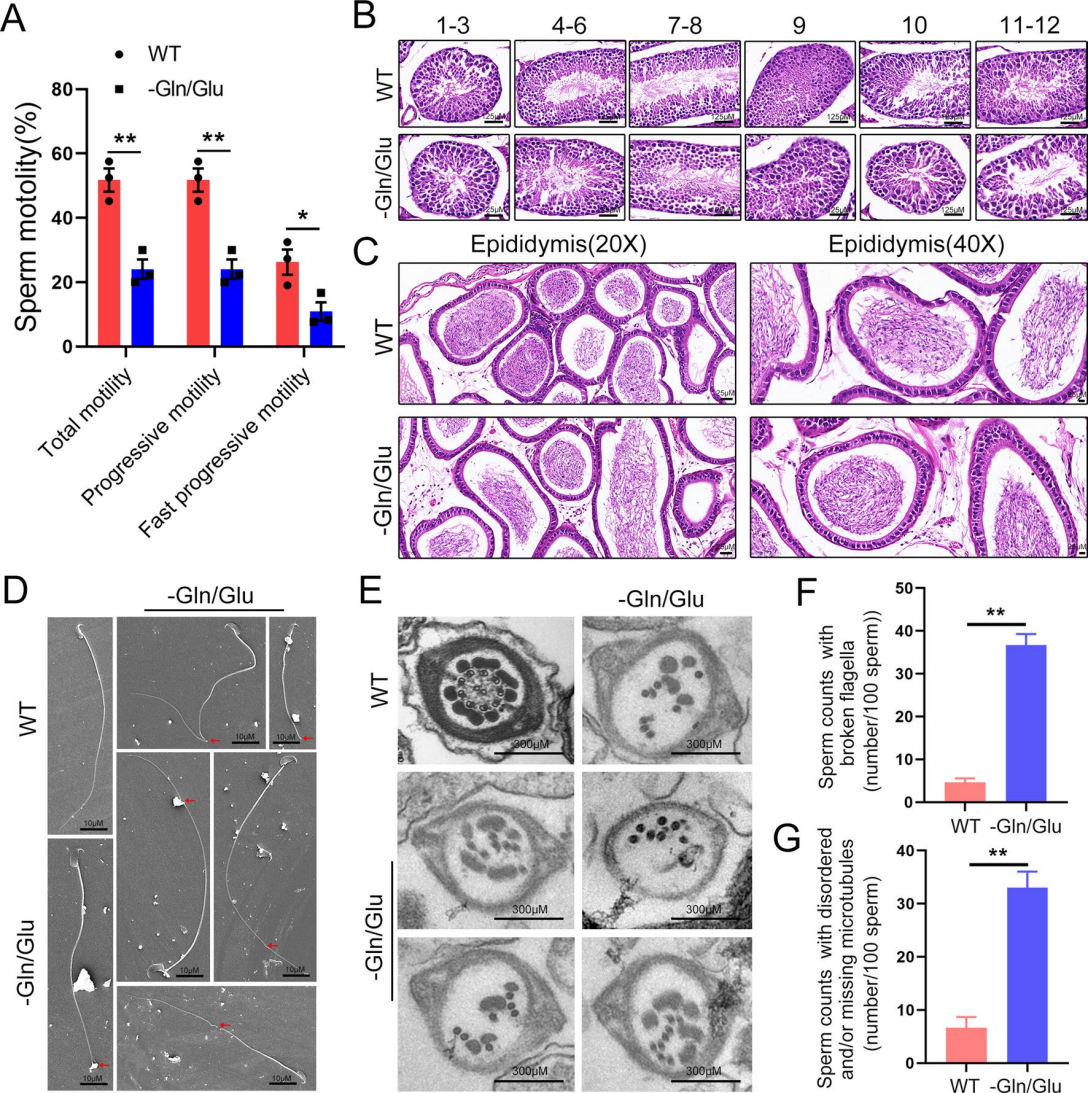
Sperm motility is decreased and the microtubule structure of the flagella is abnormal in mice under a Gln/Glu-free diet. WT, wild-type; -Gln/Glu, mice under a Gln/Glu-free diet. **A** The computer-aided sperm analysis (CASA) showed decreased sperm motility in mice with absent Gln/Glu in diets. N = 3, Student’s *t* test, *P < 0.05, **P < 0.01, error bars, s.e.m. **B** No significant abnormality of spermatogenesis was observed in seminiferous tubules of mice under a Gln/Glu-free diet. 1–12 represent the different developmental stages of the spermatogenic epithelium. Scale bars, 125 µm. **C** Normal quantities of sperm were observed in the epididymis of mice under a Gln/Glu-free diet. Scale bars, 25 µm. **D**–**G** SEM showed morphologically normal head and developed flagella in the sperm of mice under a Gln/Glu-free diet. However, numerous sperm with bent or broken flagella were observed (**D**). Scale bars, 10 µm. TEM showed disorderly arranged microtubule structure in flagella of mice under a Gln/Glu-free diet (**E**). Scale bars, 300 nm. The proportion of sperm with abnormal flagella significantly increased in mice under a Gln/Glu-free diet (**F**, **G**). N = 3, Student’s *t* test, **P < 0.01, error bars, s.e.m

Based on the clarification of the key role of QRICH2 in regulating Gln/Glu metabolism and maintaining the glutamylation levels of tubulin, we attempted to further confirm the essential role of Gln/Glu in regulating spermatogenesis and sperm motility by constructing a mouse model with absent Gln/Glu in diets. The WT male mice were fed with a Gln/Glu-free diet from postnatal 4–9 weeks. We then found a reduced motility of mice under a Gln/Glu-free diet compared with normal diets (Fig. 4a). Similar to the WT mice, the mice under a Gln/Glu-free diet displayed orderly arranged spermatogenic cells of different stages in the seminiferous tubule and showed no abnormality in quantities of elongated sperm (Fig. 4b, c). Large quantities of elongated sperm with extending flagella were observed in the seminiferous tubules of stages 7–8 (Fig. 4b). These results suggested that the Gln/Glu-free diet did not affect spermatogenesis in the seminiferous epithelium, including meiosis and spermiogenesis. However, when the ultrastructure of sperm was scrutinized under scanning electron microscope (SEM), a certain number of sperm showed a crack in the flagella of mice under a Gln/Glu-free diet, as indicated by red arrows in Fig. 4d. Meanwhile, transmission electron microscope (TEM) analysis displayed missing and/or disorderly arranged microtubule structure and ODF in flagella of mice under a Gln/Glu-free diet compared with the orderly arranged typical “9 + 2” structure in WT mice (Fig. 4e). The quantities of sperm with abnormal flagella and microtubule structures in the Gln/Glu deficiency group were higher than in the wild-type group (Fig. 4f, g). The association between these observations and the diminished glutamylation level and stability of tubulin necessitates additional scholarly inquiry to establish conclusive evidence.

### Absence of Gln/Glu in diets in mice causes decreased glutamylation levels and expression of α-TUBULIN

**Fig. 5:**
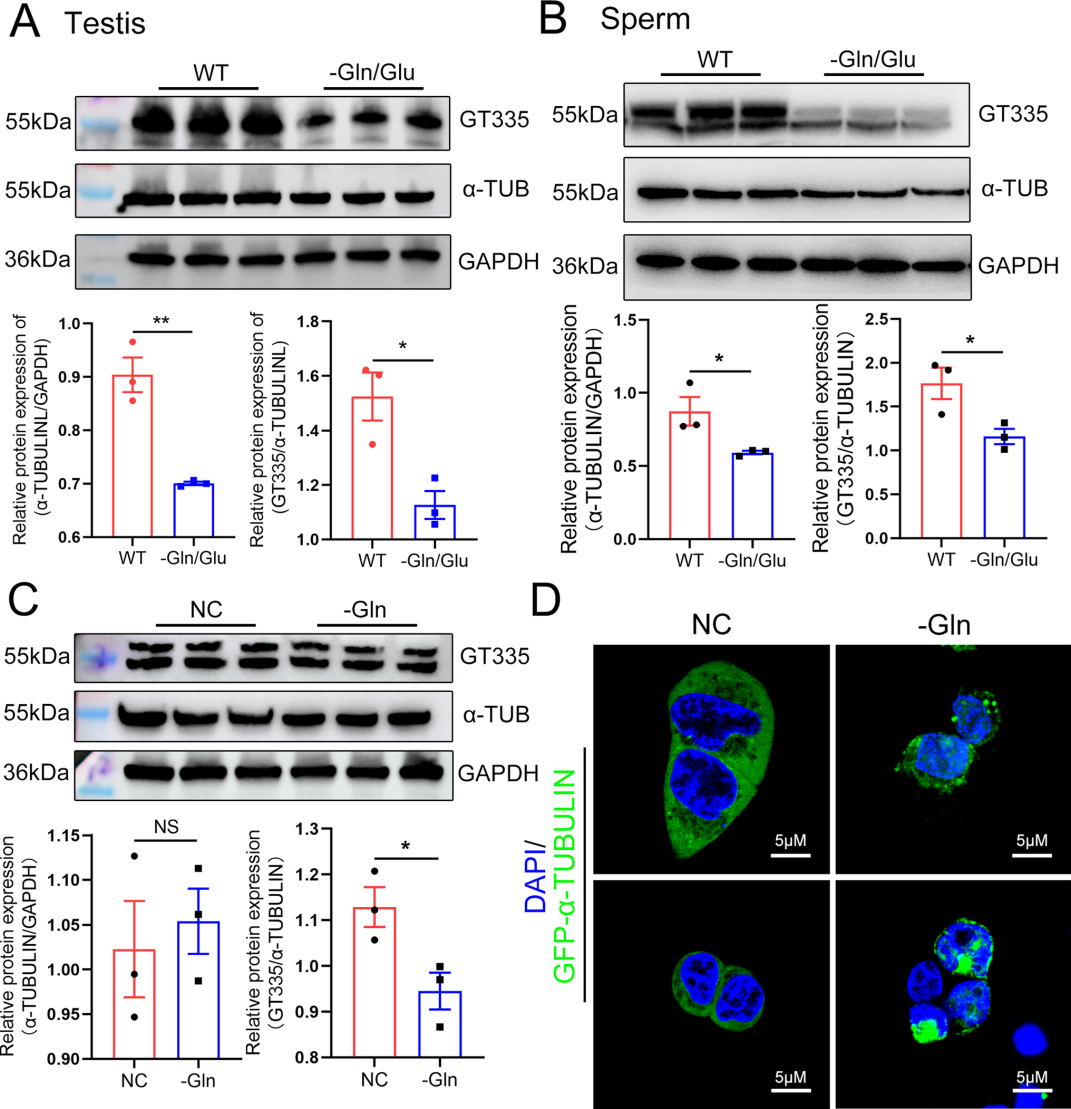
Glutamylation levels and expression of tubulin are decreased in sperm flagella of the mice with absent Gln/Glu in diets. WT, wild-type; -Gln/Glu, mice under a Gln/Glu-free diet; NC, normal control; -Gln, absence of Gln in cell culture medium. **A**, **B** Absence of Gln/Glu in the diets reduced the tubulin glutamylation levels and the α-TUBULIN expression in testes (**A**) and sperm flagella (**B**). The grayscale analysis of the protein bands was shown in the bottom panel. N = 3, Student’s *t* test, *P < 0.05, **P < 0.01, error bars, s.e.m. **C** Absence of Gln/Glu in the culture medium reduced the tubulin glutamylation levels. N = 3, Student’s *t* test, *P < 0.05, NS, *P* > 0.05, error bars, s.e.m. **D** Absence of Gln in the culture medium changed the distribution of α-TUBULIN in HeLa cells. DAPI, blue; GFP-α-TUBULIN, green; scale bars, 5 µm

**Fig. 6:**
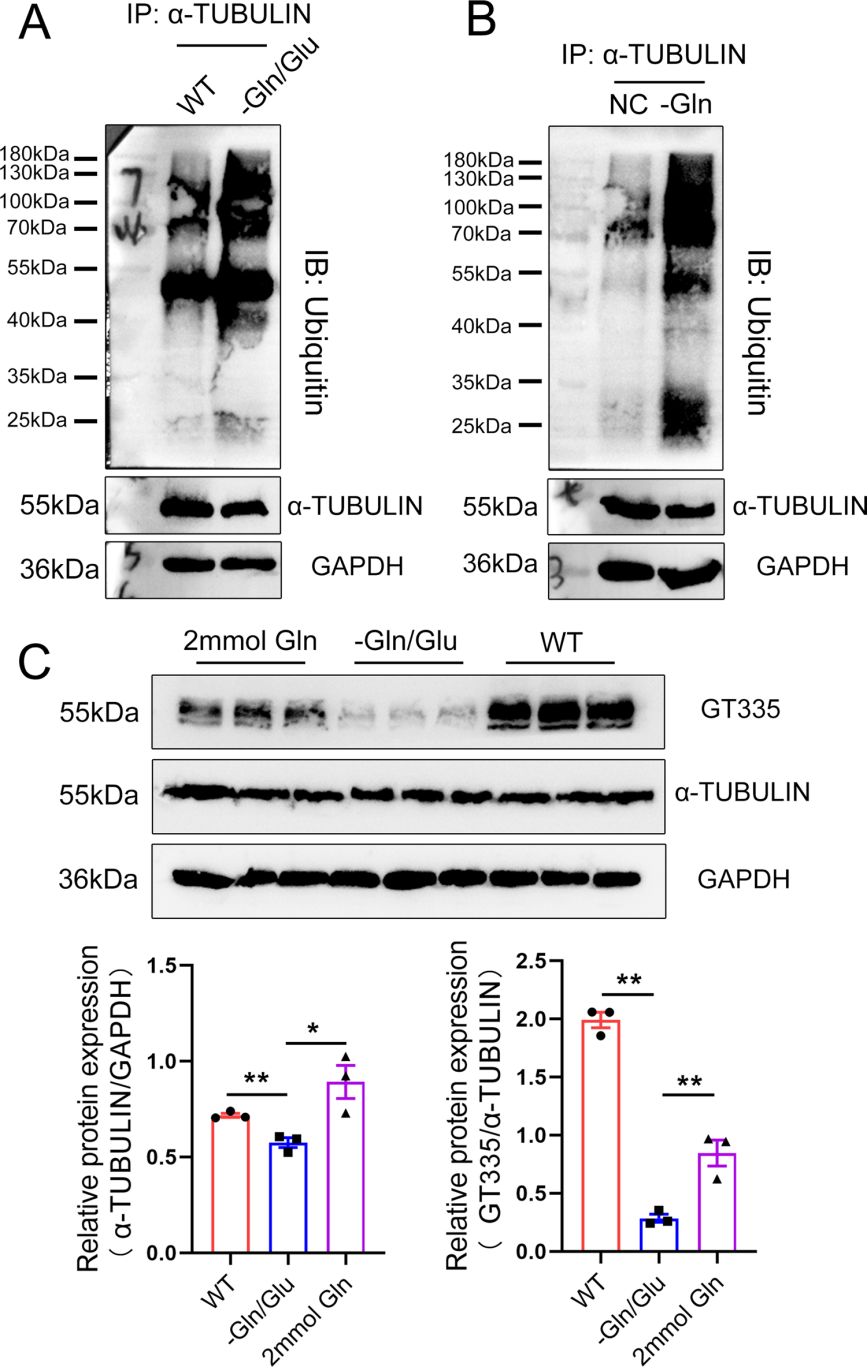
Increased ubiquitination levels of α-TUBULIN are observed in the testes of mice under a Gln/Glu-free diet. WT, wild-type; -Gln/Glu, mice with absent Gln/Glu in diets; -Gln, absence of Gln in cell culture medium. **A** Co-IP experiments showed increased ubiquitination levels of α-TUBULIN in the testes of mice under a Gln/Glu-free diet. **B** Absence of Gln in the culture medium for 72 h caused increased ubiquitination levels of α-TUBULIN in HeLa cells. **C** Addition of 2 mM Gln in vitro partially rescued the decreased glutamylation levels of tubulin induced by a Gln/Glu-free diet. The grayscale analysis of the protein bands was shown in the bottom panel. N = 3, Student’s *t* test, *P < 0.05, **P < 0.01, error bars, s.e.m

To further figure out whether the Gln/Glu are the precursors and substrates for positively regulating glutamylation levels of tubulin, we utilized the mouse model with absent Gln/Glu in diets to perform analyses. Impressively, significantly reduced glutamylation levels and expression of α-TUBULIN were observed in the testes and sperm of mice under a Gln/Glu-free diet (Figs. 5a, b, S4A), which were mediated by the decreased concentration of Gln and Glu in testes and sperm (Fig. S4B, C). At the cellular level, the absence of Gln in a culture medium for 72 h reduced the glutamylation levels of tubulin (Fig. 5c). Interestingly, in the Gln absent group, the α-TUBULIN manifested as disrupted distribution and abnormal accumulation in cytoplasm (Fig. 5d). Similar to the *Qrich2* KO mice, the ubiquitination levels of α-TUBULIN were increased in mice with absent Gln/Glu in diets and cell absence of Gln (Fig. 6a, b). When the sperm of mice under a Gln/Glu-free diet was incubated with 2 mM Gln for 2 h in vitro, the reduced glutamylation levels of tubulin were partially rescued (Fig. 6c). In addition, we observed a decreased expression of QRICH2 in the testes of mice with absent Gln/Glu in diets and cells with absent Gln (Fig. S5A–C), indicating the possibility of QRICH2 as a potential sensor of Gln/Glu concentration, functioning in Gln/Glu metabolism and regulated by Gln/Glu concentration. In conclusion, the absence of Gln/Glu in diets caused reduced glutamylation levels of tubulin in sperm, and thus increased the ubiquitination levels of α-TUBULIN and reduced the stability of α-TUBULIN.

### Mitochondria-associated protein transport and mitochondria function are impaired in sperm of *Qrich2* KO mice and mice with Gln/Glu-free diet owing to depolymerized microtubules

**Fig. 7:**
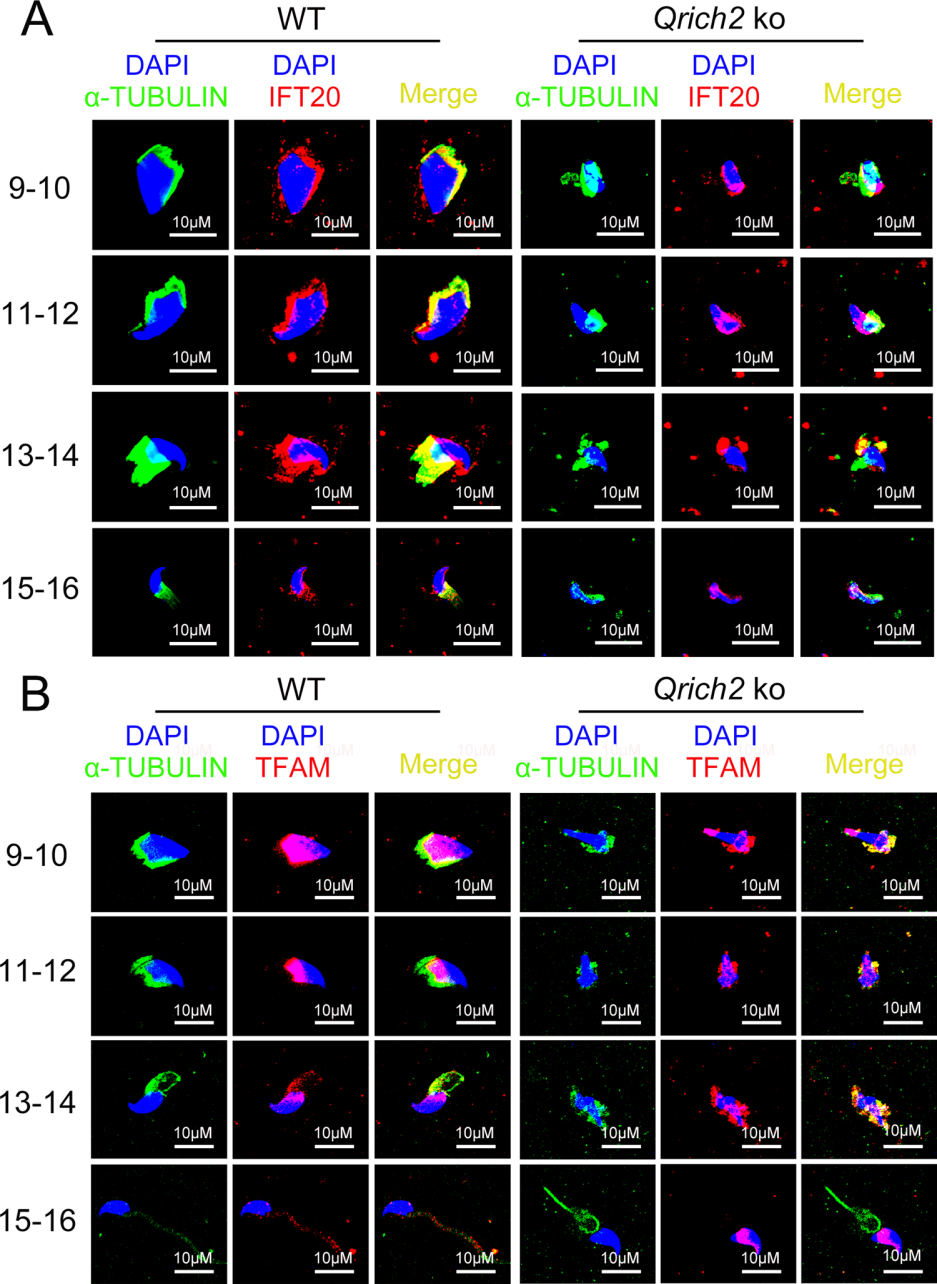
Mitochondria fail to be transported and normally distributed into flagella in *Qrich2* KO mice. WT, wild-type; *Qrich2* KO, *Qrich2* knockout. IFT20 (**A**) and TFAM (**B**) were disorderly distributed in the cytoplasm of spermatogenic cells of different developmental stages. DAPI, blue; α-TUBULIN, green; IFT20 (**A**) and TFAM (**B**), red. 9–16 represented the spermatogenic cells of different developmental stages. Scale bars, 10 µm

Mitochondrial-cytoskeleton interactions are involved in regulating mitochondrial transport and localization [18]. The abnormal mitochondrial structure observed in mice with loss of function of KLC3 and IFT20 also indicated the vital role of microtubule mediated intraflagellar transport in mitochondrial morphogenesis during spermatogenesis [37, 38]. Because our results revealed the decreased glutamylation levels and stability of tubulin in the sperm of *Qrich2* KO mice, we aimed to address whether the microtubule-based intraflagellar transport (including mitochondrial transport) was impaired. In WT mice, the expression of intraflagellar transport protein 20 homolog (IFT20) and mitochondrial component protein transcription factor A (TFAM) during spermatogenesis showed consistent temporal and spatial patterns (Fig. 7a, b). They were transported along with the developing flagella and, ultimately, TFAM was localized into the mitochondrial sheath of sperm (Fig. 7b). However, in spermatids of *Qrich2* KO mice, IFT20 and TFAM were found to be disorderly distributed in the cytoplasm (Fig. 7a, b). These results suggested that the reduced glutamylation levels and stability of tubulin in sperm of *Qrich2* KO mice disrupted the transport and localization of mitochondria components to sperm flagella which may lead to to alterations in mitochondrial function.

**Fig. 8:**
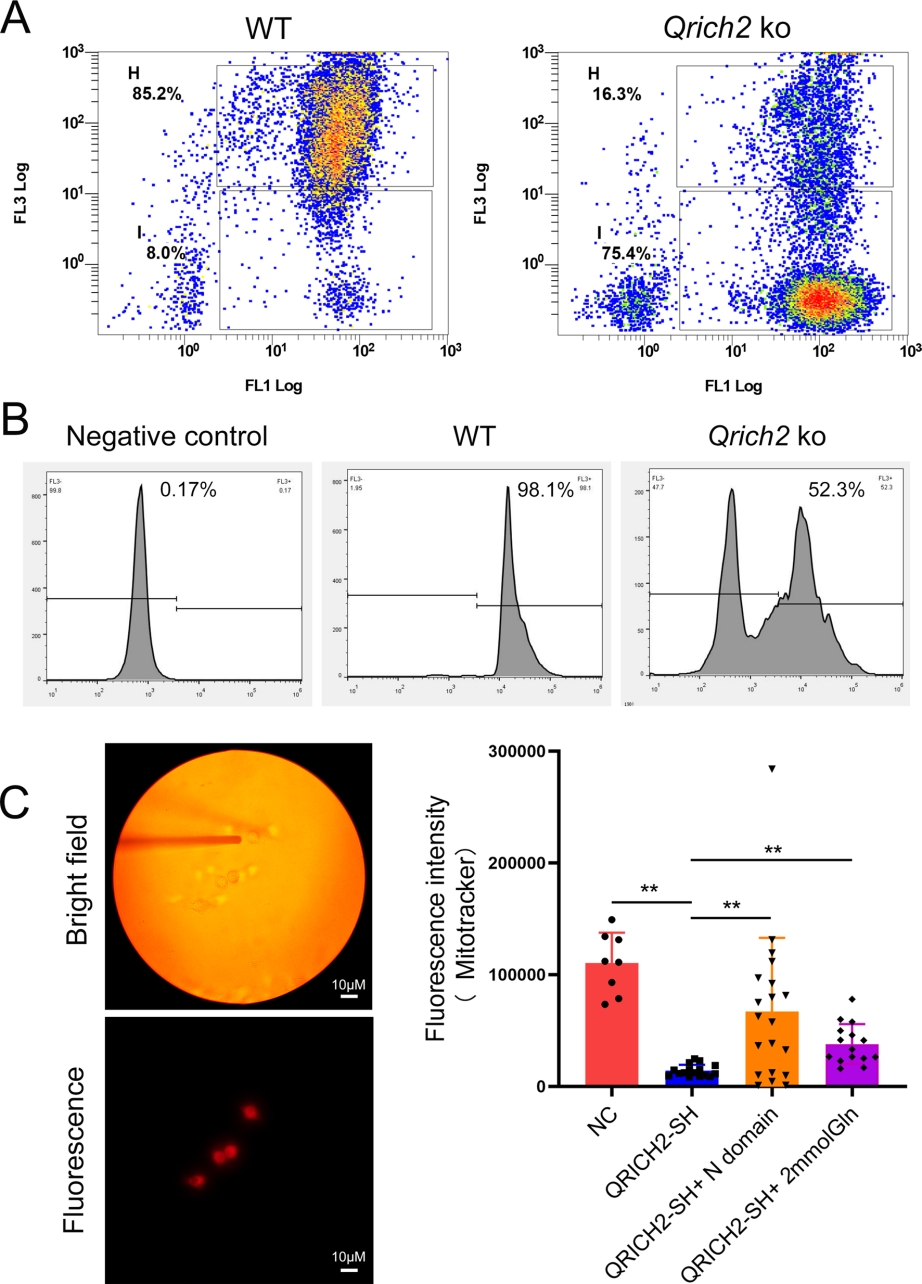
Sperm mitochondrial function of *Qrich2* KO mice is significantly decreased. WT, wild-type; *Qrich2* KO, *Qrich2* knockout; NC, normal control; QRICH2-SH, QRICH2 knockdown; N domain, QRICH2 N-terminal purified protein, 5 µg/ml. **A** JC1 staining indicated the significantly decreased mitochondrial membrane potential of sperm in *Qrich2* KO mice. The box in the upper panel displayed the cells with high mitochondrial membrane potential; the box in the bottom panel displayed the cells with low mitochondrial membrane potential. N = 3, 5000–10000 cells were analyzed for each experiment. **B** Mitotracker staining showed that sperm mitochondrial activity was significantly decreased in *Qrich2* KO mice. The horizontal axis represents the fluorescence intensity and the vertical axis represents the sperm quantity. N = 3, 5000–10000 cells were analyzed for each experiment. **C** Single cell metabolism analysis showed the detection of single cell fluorescence signal with a 5 µm light probe (left panel). Incubated with the N-terminal purified protein of QRICH2 (5 µg/ml) for 2 h in vitro partially rescued the reduced mitochondrial function caused by QRICH2 knockdown. The number of cells analyzed in each group was 8, 16, 20, and 15

Subsequently, the mitochondrial function of sperm in *Qrich2* KO mice was evaluated. Significantly reduced mitochondrial membrane potential and activity were observed in the sperm of *Qrich2* KO mice (Fig. 8a, b). The decreased levels of mitochondrial-specific reactive oxygen species (ROS) derived from the mitochondrial respiratory chain reflected the decreased mitochondrial respiratory function from the side (Figs. S6A and S6B). Consistent with the in vivo data, we found reduced mitochondrial function in cells with QRICH2 knockdown, which could be partially rescued by incubating with QRICH2 N-terminal purified protein or 2 mM Gln (Figs. 8c and S6C). In addition, the expression of several key molecules in the mitochondrial oxidative phosphorylation pathway was decreased in the testes and sperm of *Qrich2* KO mice (Fig. S7), further emphasizing the key role of *Qrich2* in maintaining the normal mitochondrial function of sperm. This preliminary validation supports our hypothesis that the reduced tubulin glutamylation and stability caused by *Qrich2* deletion may further mediate aberrations in mitochondrial transport and function.

**Fig. 9:**
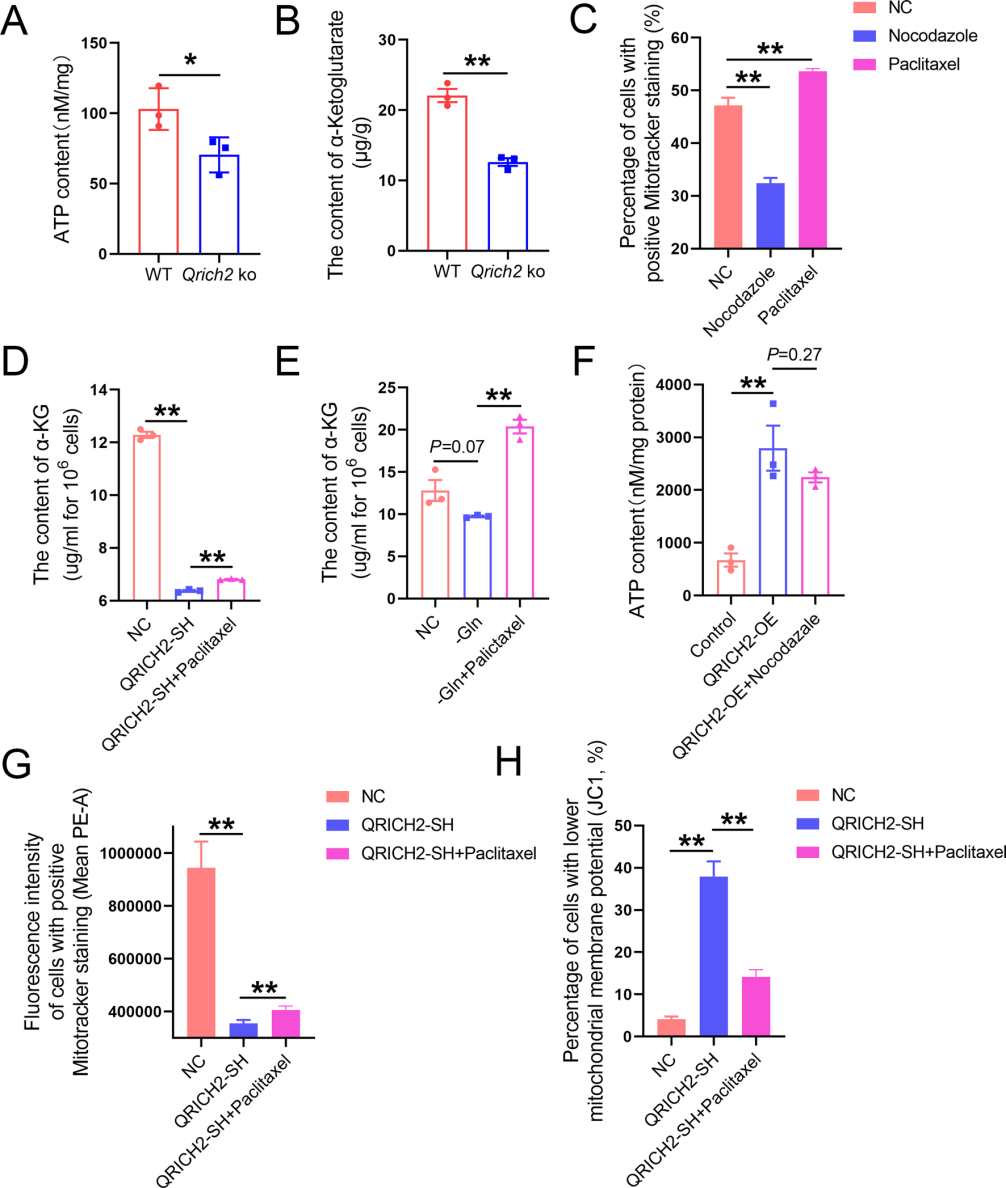
Polymerization state of microtubules is involved in regulating mitochondrial function. WT, wild-type; NC, normal control; QRICH2-OE, QRICH2 overexpression; QRICH2-SH, QRICH2 knockdown; –Gln, deficiency of Gln in culture medium. The contents of ATP (**A**) and α-ketoglutarate (**B**) were reduced in the testes of *Qrich2* KO mice compared with WT mice. **C** Nocodazole reduced the staining of Mitotracker whereas paclitaxel increased the staining. Paclitaxel partially rescued the reduced production of α-ketoglutarate induced by QRICH2 knockdown (**D**) and Gln deficiency (**E**). **F** Nocodazole inhibited the increase of ATP production caused by QRICH2 overexpression. Paclitaxel partially rescued the reduced mitochondrial function induced by QRICH2 knockdown including the reduced staining of Mitotracker (**G**) and the reduced mitochondrial membrane potential (**H**). N = 3, Student’s *t* test, *P < 0.05, **P < 0.01, error bars, s.e.m

Moreover, the mitochondrial function of sperm in mice with absent Gln/Glu from diets was evaluated and our results showed significantly reduced mitochondrial membrane potential (Fig. S8A) and mitochondrial function (Fig. S8B–D) in the mouse model. HeLa cells also displayed a significant decrease in mitochondrial function when the Gln was absent in the culture medium (Fig. S9A, B). Interestingly, the decreased mitochondrial function caused by Gln absence was partially rescued by incubating with QRICH2 N-terminal purified protein or 2 mM Gln (Fig. S9C). Similar to the findings in *Qrich2* KO mice, the expression of several key molecules in the oxidative phosphorylation pathway was reduced in the testes and sperm of mice under a Gln/Glu-free diet (Fig. S9D). As a result, the production of ATP was reduced in *Qrich2* KO mice and mice under a Gln/Glu-free diet (Figs. 9a, S10B). Alpha-ketoglutarate is a key metabolic hub molecule regulated by Glu. Therefore, we determined the level of alpha-ketoglutarate and we observed that decreased levels of Glu reduced the contents of alpha-ketoglutarate entering the citric acid cycle, which was observed in *Qrich2* KO mice and cells with QRICH2 knockdown (Fig. 9b, d). At the cellular level, overexpression of QRICH2 increased the concentration of ATP and knockdown of QRICH2 reduced the concentration of ATP (Fig. S10A). Moreover, we found that microtubule depolymerizer nocodazole reduced the mitochondrial function whereas microtubule polymerizer paclitaxel improved the mitochondrial function (Figs. 9c, S10C). Paclitaxel partially rescued the reduced mitochondrial function induced by QRICH2 knockdown and Gln deficiency, including the decrease of the alpha-ketoglutarate production, the reduced staining of Mitotracker, and the reduced mitochondrial membrane potential (Figs. 9d–h, S10D, E). In summary, our data further strengthened the connections between microtubule stability and mitochondrial function.

### Glutamylation levels of tubulin can be a metabolic biomarker in asthenospermia patients

**Fig. 10:**
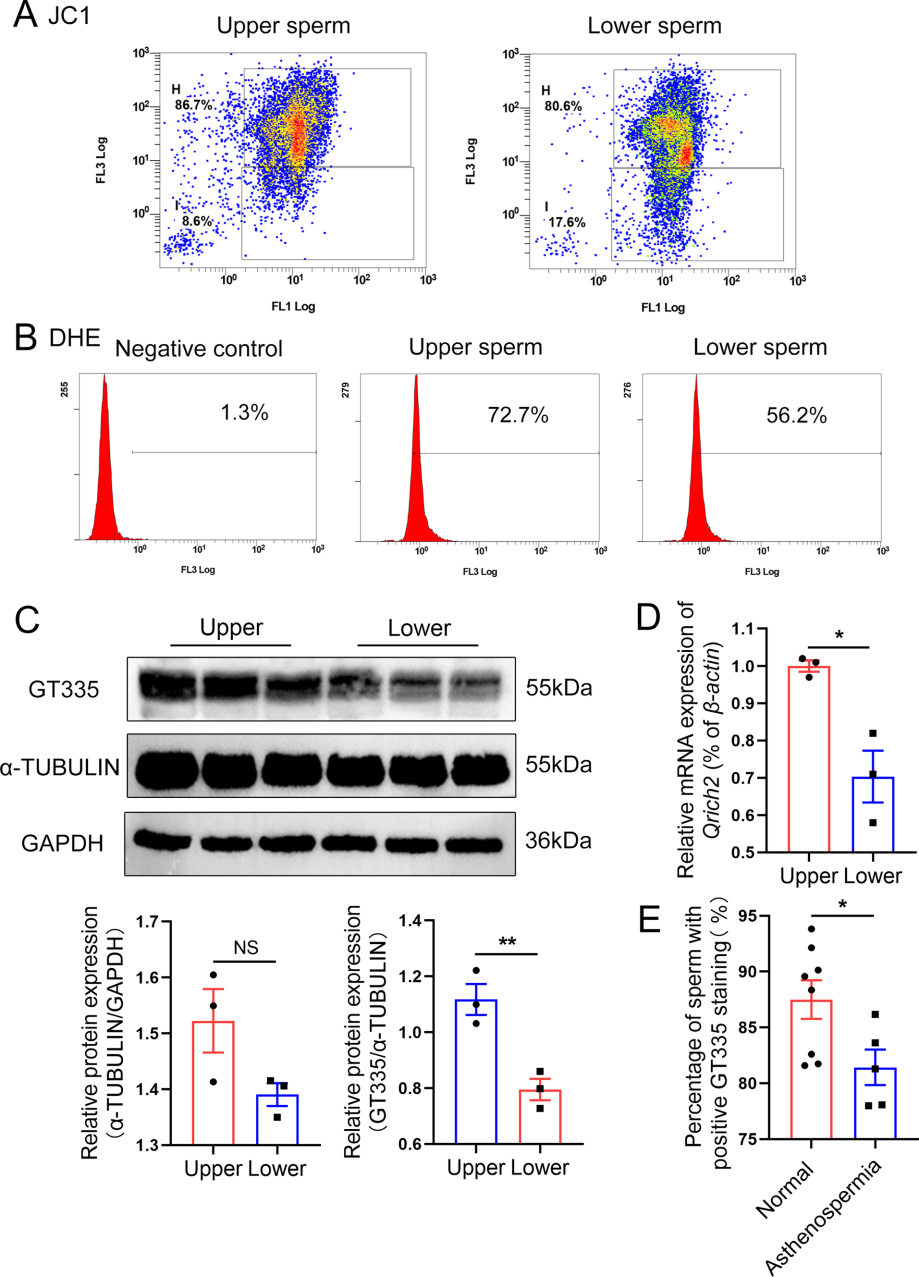
The glutamylation levels of tubulin are positively correlated with mitochondrial function and sperm motility. Upper represents the upper sperm. Lower represents the lower sperm. **A** The sperm of heterozygous *Qrich2* mice were separated by the swimming up method and the upper sperm had a higher mitochondrial membrane potential. The box in the upper panel displayed the sperm with high mitochondrial membrane potential; the box in the bottom panel displayed the sperm with low mitochondrial membrane potential. N = 3, 5000–10000 sperm were analyzed for each experiment. **B** The sperm of heterozygous *Qrich2* mice were separated by the swimming up method and the upper sperm had a higher mitochondrial specific reactive oxygen species. The horizontal axis represents the fluorescence intensity and the vertical axis represents the sperm quantity. N = 3, 5000–10000 sperm were analyzed for each experiment. **C** The sperm of heterozygous *Qrich2* mice were separated by the swimming up method and the upper sperm had a higher glutamylation levels of tubulin and higher expression of α-TUBULIN. The grayscale analysis was shown in the bottom panel. N = 3, Student’s *t* test, **P < 0.01, NS, *P *> 0.05, error bars, s.e.m. **D** The *Qrich2* expression of the upper sperm was higher than that of the lower sperm. N = 3, Student’s *t* test, *P < 0.05, error bars, s.e.m. **E** The flow-cytometry analysis of GT335 staining showed that the tubulin glutamylation levels in the sperm of patients with asthenospermia were lower than in normal controls. N = 5 for asthenospermia patients and N = 8 for normal controls

In a previous study, we showed that heterozygous *Qrich2* mice have reduced sperm motility [25]. We then used a new sperm-separating method to sort the sperm with high motility and low motility with our microfluid-based separating method. Interestingly, when the sperm from heterozygous *Qrich2* KO mice were separated into the upper and lower layers, the upper sperm with higher motility were shown to have a better mitochondrial function (Fig. 10a, b). Furthermore, our results showed that the upper sperm displayed higher glutamylation levels of tubulin (Fig. 10c) and expression of *Qrich2* (Fig. 10d), and the patients with asthenospermia manifested a lower level of tubulin glutamylation in sperm (Fig. 10e). The positive correlations between higher *Qrich2* expression and tubulin glutamylation levels, better mitochondrial function, and higher sperm motility were established and further supported the notion that the Gln sensing function of *Qrich2* plays a critical role in regulating tubulin glutamylation, mitochondrial function, and sperm motility in mice and humans.

## Discussion

Our previous study identified two nonsense mutations of *QRICH2* in infertile patients from two consanguine families that caused degradation and loss of function of the QRICH2 protein. The sperm of the patients manifested as typical MMAF phenotype [25]. We then generated the *Qrich2* KO mice and found similar sperm phenotype to the patients, i.e., the KO mice were infertile. Unexpectedly, the present study revealed the key role of QRICH2 in regulating Gln/Glu metabolism and maintaining tubulin stability in the testis and sperm. QRICH2 positively regulated the glutamylation levels of tubulin and maintained its stability by promoting the conversion of Gln to Glu. Similar to the findings in *Qrich2* KO mice, we observed decreased Gln/Glu concentration, reduced glutamylation levels and stability, and increased ubiquitination levels of α-TUBULIN in testes and sperm of mice under a Gln/Glu-free diet. These results further emphasized the significance of the QRICH2 as a Gln sensor involved in regulating tubulin glutamylation levels and stability in the assembly of flagella.

Mechanistically, our results indicated that QRICH2 might regulate the tubulin glutamylation and function by interacting with tubulin protein. Our IP-Mass spectrometry results showed that QRICH2 could interact with TUBA1C, TUBB4B, and TUBB, and our Co-IP results further confirmed the interaction between QRICH2 and α-TUBULIN. Gene expression results showed that QRICH2 regulates the expression of glutamine synthase GLUL and glutaminase GLS, and deletion of *Qrich2* caused increased expression of *Abgl5* (*Ccp5*) and down-regulation of *Ttll4*. A recent study showed that a tubulin glutamylation regulator could function as a protein complex regulating tubulin glutamylation and controlling cell nucleus morphology and other processes [34]. Consequently, further investigation is needed on whether QRICH2 controls tubulin glutamylation modification by directly interacting with regulators of glutamylation including TTLLs and CCPs, in addition to functioning as a complex. Interestingly, a recent paper also showed that deletion of TTLL5 causes sperm malformation [16]. Compared with the previous study of the TTLL5’s function in spermatogenesis, both the loss function of *Ttll5* and *Qrich2* caused reduced glutamylation levels and abnormal structure of tubulin. However, the KO of *Qrich2* demonstrated much more serious impairment to spermatogenesis than the *Ttll5* KO, manifested as more severe flagella deformities, less sperm quantity, and complete loss of fertility, suggesting the wider role of QRICH2 in spermatogenesis. Considering our previous results that QRICH2 regulated the ubiquitination of known key proteins for flagella development, we suggest the decreased tubulin glutamylation and stability caused by *Qich2* KO partially contribute to the abnormal flagella development and sperm mitochondrial functional regulation.

Except for regulating flagella development, *Qrich2* also plays a role in maintaining normal mitochondrial function. We have confirmed that *Qrich2* KO/knockdown reduced the mitochondrial function of sperm/cells at both animal and cell levels, thus causing decreased ATP production, and similar results were observed in mice with absent Gln/Glu in diets. Considering the mitochondrial sheath location in the outermost layer of the middle piece [11], the dysplasia of flagella, especially the short tail and curly tail, usually leads to an abnormal mitochondrial structure. The mitochondrial-cytoskeleton interaction interface in mammals is conserved at the molecular levels and the function is involved in the regulation of the transport, localization, and anchoring of mitochondria [5, 10, 18, 21, 31, 32]. Thus, the correct assembly and stability of microtubule-based cytoskeleton structure are essential in intraflagella transport, including mitochondria [23, 38]. In the present study, we found disordered distribution and traffic of IFT20 and mitochondrial component protein TFAM in spermatids of developmental stages; this indicated that the decrease in tubulin glutamylation levels and stability in spermatids of *Qrich2* KO mice affected the intraflagellar cargo transportation, thus being involved in mediating the abnormalities of mitochondrial function and structure. In the sperm of mice with absent Gln/Glu in diets, the mitochondrial function was significantly decreased, which, induced by Gln absence, was partially rescued by 2 mM Gln in vitro. These results suggested that the reduced mitochondrial function caused by *Qrich2* KO is not only caused by the abnormal mitochondrial transport and localization but also due to the reduced levels of Gln which caused a decrease of α-Ketoglutarate entering the TCA cycle. Moreover, paclitaxel partially rescued the reduced mitochondrial function induced by QRICH2 knockdown and Gln deficiency, strengthening the connections between reduced mitochondrial and QRICH2 disruption-induced microtubule depolymerization.

In clinical practice, the swimming-up method is usually used to screen sperm with better motility for in vitro fertilization to improve the fertilization rate. Interestingly, after the sperm from *Qrich2* heterozygous mice was sorted into the upper and lower layers by the swimming-up method, we found higher glutamylation levels of tubulin in sperm of the upper layer with better motility, indicating the potential role of tubulin glutamylation levels as a new target for screening sperm with better motility for clinical practice. Using tubulin glutamylation antibody GT335, we performed flow cytometry to detect the glutamylation levels of sperm in men with normal fertility and patients with asthenospermia and found reduced glutamylation levels in sperm of asthenospermia patients. Thus, it is possible that a tubulin glutamylation marker, such as the GT335 antibody, could be used in flow cytometry as a new cost-effective method for clinical sperm screening. The N-terminal purified protein of QRICH2 and 2 mM Gln could partially rescue the reduced tubulin glutamylation levels and mitochondrial function caused by *Qrich2* KO and Gln absence, which suggested the QRICH2 N-terminal purified protein and Gln as the potential cure method for treating the asthenospermia patients with the reduced tubulin glutamylation levels.

Although our study emphasized the importance of tubulin glutamylation levels and stability in sperm flagella assembly and intraflagella transport, there are still several limitations that need to be solved in future research. First, the metabolomics results showed no significant alteration in the concentration of Glu in the testes, while the concentration was decreased by in vitro detection. A discrepancy was found because of the heterogeneity of cell types in testes, including somatic cells and spermatogenic cells of different stages. Thus, the results in the testes did not accurately reflect the Gln/Glu levels of sperm. We subsequently detected the Glu levels of sperm in vitro and a reduced concentration was observed in sperm similar to that of testes from *Qrich2* KO mice. Second, we found that the purified N-terminal protein of QRICH2 in vitro partially rescued the reduction of tubulin glutamylation levels and tubulin stability caused by *Qrich2* KO, but the mechanism was not completely clear. We speculated that the N-terminal purified protein of QRICH2 was involved in regulating Gln/Glu metabolism or its decomposition in the process of incubating with sperm directly affected the concentration of Gln/Glu. Moreover, we observed a significant decrease in tubulin glutamylation levels in the sperm of *Qrich2* KO and mice under a Gln/Glu-free diet, whereas the expression of α-TUBULIN was just slightly decreased, indicating that the glutamylation levels of tubulin were not the only factor affecting tubulin stability; there may be other ways or post-translational modifications playing a role in regulating tubulin stability. Lastly, the observation of decreased expression of *Qrich2* in the testes and sperm of mice under a Gln/Glu-free diet indicated a close relationship between *Qrich2* and Gln/Glu metabolism. However, the exact mechanism of how *Qrich2* senses the alteration of Gln/Glu concentration to regulate the downstream pathway warrants further exploration.

In conclusion, through experiments at animal and cellular levels, we revealed how the deletion of *Qrich2* in mice affects tubulin stability during spermatogenesis through sensing Gln/Glu metabolism in testes and sperm. First, the glutamylation levels and stability of microtubules were reduced, failing flagella assembly. Second, the microtubule structure-based intraflagellar transport was attenuated, thus affecting the distribution and localization of mitochondria. Both factors eventually led to reduced sperm motility and infertility in male mice. Our results demonstrated that QRICH2 acted as a possible novel glutamine sensor linking Glu/Gln metabolism, tubulin glutamylation, and male fertility.

## Materials and methods

### Animal breeding and model construction

All of the animal experiments were conducted in accordance with the guidelines for the care and use of laboratory animals formulated and implemented by the National Institutes of Health. The animal experiments were approved by the experimental animal management and ethics committee of West China Second Hospital. All of the animals were kept in specific pathogen free (SPF) animal rooms for 12-h day–night alternation, under a standard animal diets and with free access to water. The details for generating *Qrich2* KO mice were described in a previous study [25]. For comparing the effect of the diets, 12 male C57BL/6 J mice aged 4 weeks were randomly divided into two groups and fed with a control diets or Gln/Glu-free diets for 5 weeks. The specific feed ingredients are shown in Table S1.

### Participants involved in the study

Eight healthy Han males with normal fertility and five patients clinically diagnosed with asthenospermia were recruited in this study. The present study was approved by the Institutional Ethics Committees of West China Second Hospital. The study conforms with the Helsinki Declaration of 1975 (as revised in 2008) concerning Human and Animal Rights. All of the participants in this study signed the informed consent.

### Quantitative polymerase chain reaction

Briefly, total RNA was extracted from tissue and cell samples using TransZol Up Plus RNA Kit (TransGen Biotech, ER501-01). Subsequently, the total RNA was reverse transcribed into double-stranded cDNA using SuperScript™ IV Reverse Transcriptase (Thermo Fisher, 18090010). Then the qPCR was performed in a total reaction volume of 10 µL containing 5 µL SYBR Green Mix (KCQS00, Sigma Aldrich), 3.6 µL DEPC-H_2_O, 0.2 µL forward primer, 0.2 µL reverse primer, and 1 µL cDNA (Total amount: 10 ng-100 ng). A two-step method was used: denaturation temperature of 95 °C for 30 s, annealing and extension of 60 °C for 30 s. The details were previously described [36]. The primers are shown in Tables S2 and S3.

### Hematoxylin–eosin (HE) staining

The fixed testis tissues were embedded in paraffin and then cut into 5 µm ultrathin sections. The sections were then dewaxed in dimethylbenzene and dyed in hematoxylin for 50 s. After that, the sections were placed under flowing water for 5–10 min. Subsequently, the sections were immersed into 1% HCL 2–4 times and washed under flowing water as before. Next, the sections were immersed in eosin for 10 min and then dehydrated in gradient ethanol of 70, 80, 90, and 100%. Lastly, the stained sections were dried in a bake oven and then sealed with neutral balsam for subsequent observations.

### Immunofluorescence staining

For staining of sperm and spermatogenic cells at different stages, the samples were first fixed in 4% paraformaldehyde and then homogeneously smeared onto the slides. Subsequently, the slides were punched in 0.5% Triton X-100 (Beyotime, P0096-100 m) for 10 min and then blocked in 5% BSA for 30 min at room temperature. After washing with phosphate buffered solution (PBS) twice, the slides were incubated overnight with primary antibodies (shown in Table S4) at 4 °C and then incubated with corresponding secondary antibodies at room temperature for 60 min. Subsequently, the slides were sealed with 4′,6-diamidino-2-phenylindole (DAPI, Beyotime, P0131) and observed under a laser confocal microscope (Olympus, FV1000).

For staining of testicular sections, the prepared 5 µm paraffin sections were dewaxed in dimethylbenzene and then immersed in sequential gradient ethanol (100, 100, 95, 85, 75, 50%) and distilled water. After the antigen repair in citrate sodium, the sections were then blocked in goat serum (Thermo Fisher Scientific, R37624) for 60 min at room temperature. Subsequently, the sections were successively incubated with primary antibodies overnight at 4 °C and secondary antibodies for 60 min at room temperature. After that, the sections were sealed with 5 µl DAPI and observed under a laser confocal microscope (Olympus, FV1000). The primary and secondary antibodies used in our research are shown in Table S4.

### Isolation of single spermatogenic cell from mouse testis

The mice were executed and the fresh testes were isolated and placed in PBS. Subsequently, the albuginea was cut and removed. The testis tissues were then transferred to a 1.5 ml centrifuge tube containing 0.5 ml PBS and were completely sheared. Next, the tissue debris suspension passed through a 40 µm filter screen to obtain the single spermatogenic cells. The cell suspension was then centrifuged to obtain the precipitation of spermatogenic cells and the supernatant was discarded. The single spermatogenic cells were then fixed in 4% paraformaldehyde for subsequent experiments.

### Flow cytometry of JC1, mitotracker, mitosox, and DHE staining

All of the detections were performed according to the operator’s instructions of JC1 (Beyotime, C2006), Mitotracker (Beyotime, C1049), Mitosox (Thermo Fisher, M36008), and DHE (Keygen, KGAF09) assay kits. Briefly, the living sperm or cells were stained with a working solution according to the corresponding concentration at 37 °C. After being washed twice with PBS, the samples were resuspended in a flow tube and then detected on a flow cytometer according to the corresponding excitation and emission wavelength.

### Scanning electron microscopy and transmission electron microscopy

For SEM, fresh sperm isolated from cauda epididymis of mice were washed three times using PBS and then fixed on coverslips with 4% Paraformaldehyde for 2 h. Subsequently, the coverslips carrying spermatozoa were washed with PBS and then dehydrated with gradient ethanol of 50, 60, 70, 80, 95, and 100%. Then, the sperm were dried with Quorum K850 Critical Point Dryer (East Sussex, UK) and coated with gold particles using an ion sputter coater (Rotary Pumped Quorum Technologies, Q150RS). The spermatozoa were finally observed under SEM (Hitachi, S-3400N).

For TEM, testis tissues were isolated from mice and then fixed with 2.5% glutaraldehyde for more than 24 h. Subsequently, the samples were further fixed with 1% osmic acid for 120 min. After washing with PBS twice, the samples were dehydrated with gradient acetone of 50, 70, 90, and 100% at 4 °C and then embedded in embedding agents containing 6% butylene phthalate, 1% phenol, 44% dodecyl succinic anhydride, and 56% epoxy resin. Next, ultrathin sections (70–90 nm) were obtained and double-stained with lead citrate and uranyl acetate. Finally, the images were taken under transmission electron microscopy (Philips, TECNAI G2 F20) for further analysis.

### Western blot

The details for western blot assay were previously described [36]. Briefly, collected cells and tissue samples were lysed on ice in RIPA lysis buffer for 60 min (spermatozoa lysed overnight at 4 °C) (Beyotime, P0013B), which contained the Protease Inhibitor Cocktail (Biomake, B14012). Subsequently, protein concentrations of each sample were determined by the BCA method (Solarbio, PC0020). Next, the same amounts of total proteins from 30 to 60 µg were loaded into each sampling hole and were separated via electrophoresis by 7.5–12.5% SDS-PAGE (Shanghai Epizyme Biomedical Technology Co., Ltd, PG112, PG113) and then transferred to polyvinylidene difluoride membranes (Millipore, ISEQ00010, IPVH00010). Subsequently, the membranes were successively incubated with primary antibodies overnight at 4 °C and secondary antibodies for 60 min at room temperature. Finally, the protein bands were visualized with ECL HRP substrate (Millipore, WBKLS0500). The GAPDH is used as the loading control to ensure that the total protein contents are consistent in each sampling well. The primary and secondary antibodies used in our research are shown in Table S4.

### Co-Immunocoprecipitation

The detailed methods were previously described. In short, the extracted total proteins from the mouse testes were incubated with 5–10 µl target antibodies on the rotary shaker overnight at 4 °C. Subsequently, 50 µl of Protein A/G magnetic beads (Thermo Fisher, 88804) were added into each sample and incubated for 60 min at room temperature. After washing twice and resuspension in PBS, the proteins were eluted from the beads by denaturation in 1xSDS (Biosharp, BC502A) at 95 °C for 15 min. The obtained proteins were used for further western blot analysis.

### Cell culture

HEK-293 T and HeLa cells were obtained from American type culture collection (ATCC) and cultured with DMEM (GIBCO, 11965092) containing 10% fetal bovine serum (Sigma, F8318) and 0.1% penicillin/streptomycin in an incubator with 5% CO_2_ at 37 °C. According to the experimental scheme, the overexpression (Abm, 382720940395) and knockdown (Bio-Atom Biotechnology, EV1420) plasmids of QRICH2 were transfected into the cells. After 48–72 h, the cells were collected to verify the overexpression or knockdown efficiency and conduct subsequent analysis. Furthermore, cells were cultured in the medium with absent Gln and collected for further assay after 72 h.

### Determination of the concentrations of Gln and Glu

The concentrations of Gln and Glu were determined by colorimetry according to the users’ instructions (Suzhou Grace Biotechnology Co., Ltd, G0429W, G0427W). Gln/Glu were first freed by the lysis of the mouse testis tissues or spermatozoa. Subsequently, the kit ingredients and the free Gln/Glu were mixed in the tubes to produce a color reaction and then the absorbance was recorded by the Microplate Reader according to the maximum absorption wavelength (MOLECULAR DEVICES. CmaxPlus). Finally, the Gln/Glu concentrations were calculated based on the standard curve.

### Amino acid metabonomic analysis

Metabonomic analysis was performed according to the standard experimental process.

The free amino acid mixtures were isolated from the testis tissues and then analyzed by ultra high performance liquid chromatography (Shimadzu, Nexera X2 LC-30AD) and mass spectrometry (AB SCIEX, 5500QTRAP). Subsequently, the chromatographic peak area and retention time were extracted by MultiQuant software and the 20 amino acid standards were used to correct the retention time and identify the metabolites. Two groups including WT and *Qrich2* KO were analyzed and equal mixed samples of different samples were prepared to ensure the quality control and stability of the analysis in this study.

### Microtubule polymerization experiments

The microtubule polymerization experiments were conducted as previously described [14]. The purified α-TUBULIN (HTS03) and GTP stock (BST06-1) were obtained from Cytoskeleton. The reactions were performed in 384 well plates in a volume of 50 µl. The General Tubulin Buffer (containing 4 mg/ml α-TUBULIN) based reaction systems of blank control, positive control, and sample control group were added into the plate and 1 mM GTP (final concentration) was added to prime the reactions. Immediately, the plate was placed into the preheated Microplate Reader to record the results. The absorption wavelength was set at 340 nm and the absorbance was recorded every 15 s. Paclitaxel (AbMole Bio science, 33069-62-4) at a 10 µM concentration was used as a positive control for promoting microtubule polymerization.

### Single cell metabolic analysis

The analysis was performed according to the operator's manual. The cells were prepared according to the experimental requirements. Subsequently, the cells were digested and inoculated on the confocal dish at a density of 10%. On the second day, the Mitotracker staining was conducted as previously described and then the confocal dish was placed in the Single Cell Analyzer TM (JiangSu RayMe Biotechnology Co., Ltd, SCA500). Next, the focal length and the position of the light probe were adjusted to focus on a single cell to detect the fluorescence intensity.

### Separation of sperm from *Qrich2* heterozygous mice using the upstream method

First, the cauda epididymis was separated from the heterozygous mice and then placed in the 1.5 ml EP tube containing 1 ml TYH (Easy check, M2030). An incision was produced to enable the swimming out and capacitation of the spermatozoa. Next, the tube was vertically placed in the incubator at 37 °C for 60 min. Subsequently, the 300-µl upper and lower sperm suspension were sucked out using a pipettor.

The sperm suspensions were then centrifuged at 2000 rpm for 5 min to obtain the sperm for further analysis.

### The enriched ontology clusters analysis

The enriched ontology clusters analysis was performed using the online tool Metascape (http://metascape.org). Submitting the gene list and selecting the appropriate species to be aligned for analysis.

### The construction of the phylogenetic tree of QRICH2

Twenty-one homologous genes of *QRICH2* were obtained from 12 species, including *Acipenser ruthenus*, *Rhacophorus reinwardtii*, *Xenopus laevis*, *Anolis carolinensis*, *Chrysemys picta*, *Thamnophis elegans*, *Anas platyrhynchos*, *Taeniopygia guttata*, *Melopsittacus undulate*, *Ailuropoda melanoleuca*, *Homo sapiens*, and *Mus musculus* (As shown in Table S5). Because the C16orf96 protein has a similar domain of DUF4795 with QRICH2, we set C16orf96 as an out group of the phylogenetic tree. We performed multiple alignments using Multiple Sequence Comparison by Log-Expectation, conducted the alignment curation using Cleaning aligned sequences, and finally constructed the phylogenetic tree using a method of Maximum likelihood-based inference of phylogenetic trees with Smart Model Selection. Moreover, we used the NCBI database to label the conserved domains of the obtained QRICH2 and C16orf96 proteins and merged the phylogenetic tree with the domain.

### Duolink proximity ligation assay

The PLA assay was conducted using the Duolink In Situ reagents (DUO92008, Sigma) according to the manual instructions. First, sperm cells were fixed with 4% PFA on the slides and permeabilized with 0.3% Triton X-100 in PBS for 20 min. Then the slides were blocked with 10% goat serum for 1 h at room temperature and incubated with rabbit anti-QRICH2 and mouse anti-α-TUBULIN primary antibodies. Later, the oligonucleotide-labeled anti-rabbit and anti-mouse PLA probes were used to label the primary antibodies. If the protein–protein interactions in situ are within a 40 nm distance, a PLA red fluorescent signal is generated. Subsequently, the slides were washed with PBS and mounted using anti-fade mounting media with DAPI. The images were captured using a laser confocal microscope (Olympus FV3000).

### Protein structure prediction and protein–protein docking

Three-dimensional structural models of QRICH2 and TUBULIN alpha-3 chain were downloaded from the AlphaFold Protein Structure Database [12, 30] (https://alphafold.com/). Flexible docking between QRICH2 and TUBULIN alpha-3 chain was performed on the HADDOCK webserver (https://bianca.science.uu.nl/haddock2.4/submit/1) [29]. To define the ambiguous interaction restraints (AIRs) in docking, the active and passive residues of QRICH2 and TUBULIN alpha-3 chain were predicted by the CASTp webserver (http://sts.bioe.uic.edu/castp/calculation.html) [26]. The first complex model with the lowest HADDOCK score was chosen to be the final model. And the protein–protein interaction was analyzed by LIGPLOT [33]. The structure diagram was made by PyMOL software. Furthermore, conserved motifs were predicted by MEME (Multiple EM for Motif Elicitation) motif discovery tool (http://meme-suite.org/tools/meme) [2].

### Detection of cellular ATP levels

ATP levels in sperm, testis, and cell lysates were measured using the luminometer method according to the manufacturer's instructions (S0027, Beyotime Biotechnology). For normalization, total proteins were extracted from samples before ATP assay.

### The detection of cellular α-ketoglutarate levels

The concentration of α-Ketoglutarate in samples was detected by high-performance liquid chromatography method following the manufacturer’s instructions for α-Ketoglutarate HPLC Assay kit (BC4704, Solarbio Science & Technology Co., Ltd., Beijing, China).

### Statistical analysis

Statistical analyses were performed using GraphPad Prism 8.4.0 software and SPSS 17.0 software. All of the data were presented as the means ± SEM. *P* < 0.05 was considered to be statistically significant. Statistical significance between the two groups was calculated using an unpaired, parametric, two-sided Student’s *t*-test.

### Supplementary Information

Below is the link to the electronic supplementary material.

Supplementary file 1 (DOCX 5756 kb)

## Data Availability

The data analyzed during this study are included in this published article and the supplemental data files. Additional supporting data are available from the corresponding authors upon reasonable request.
