# Supplementary material for: Metabolic profiling identifies Qrich2 as a novel glutamine sensor that regulates microtubule glutamylation and mitochondrial function in mouse sperm
Source: Cell Mol Life Sci. 2024 Apr 10;81(1):170. doi: 10.1007/s00018-024-05177-4 (PMC11006759; doi:10.1007/s00018-024-05177-4)
Supplement: Supplementary file 1 — (DOCX 5756 kb) [file 18_2024_5177_MOESM1_ESM.docx]

**Supplementary information**

**Supplementary Figures**

**
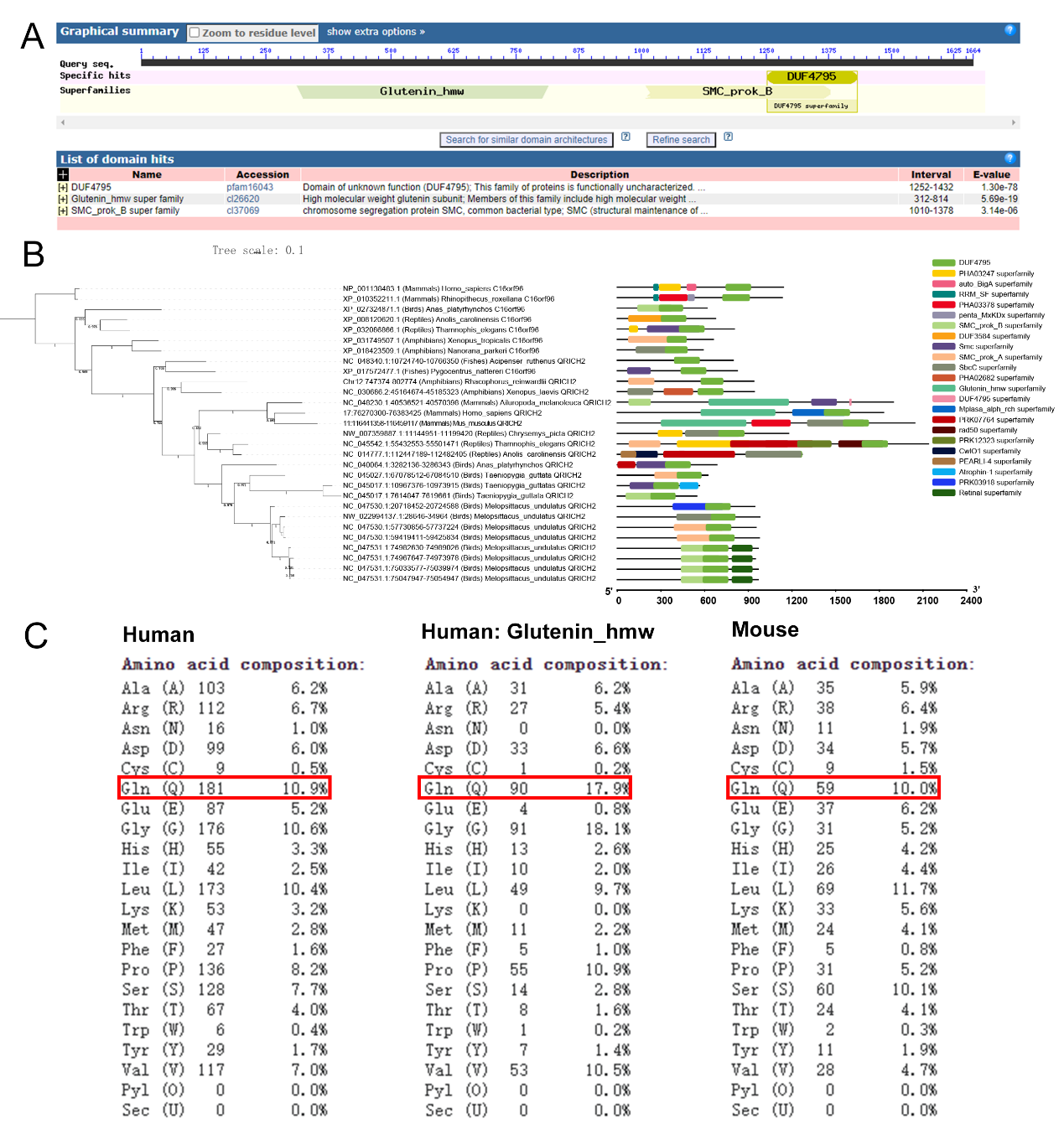
**

**Figure S1. The construction of the phylogenetic tree of QRICH2 and the annotation of the conservative domains.** (A) QRICH2 of homo sapiens contains three conservative domains including DUF4795, Glutenin_hmw and SMC_prok_B. (B) The phylogenetic tree of QRICH2 with the annotation of the conservative domains. The DUF4795 domain was highly conservative across species and the Glutenin_hmw domain appeared in mammals. (C) The QRICH2 protein, in particular the Glutenin_hmw domain was rich in Glutamine. The proportion of Gln was indicated by hollow red rectangle.

**
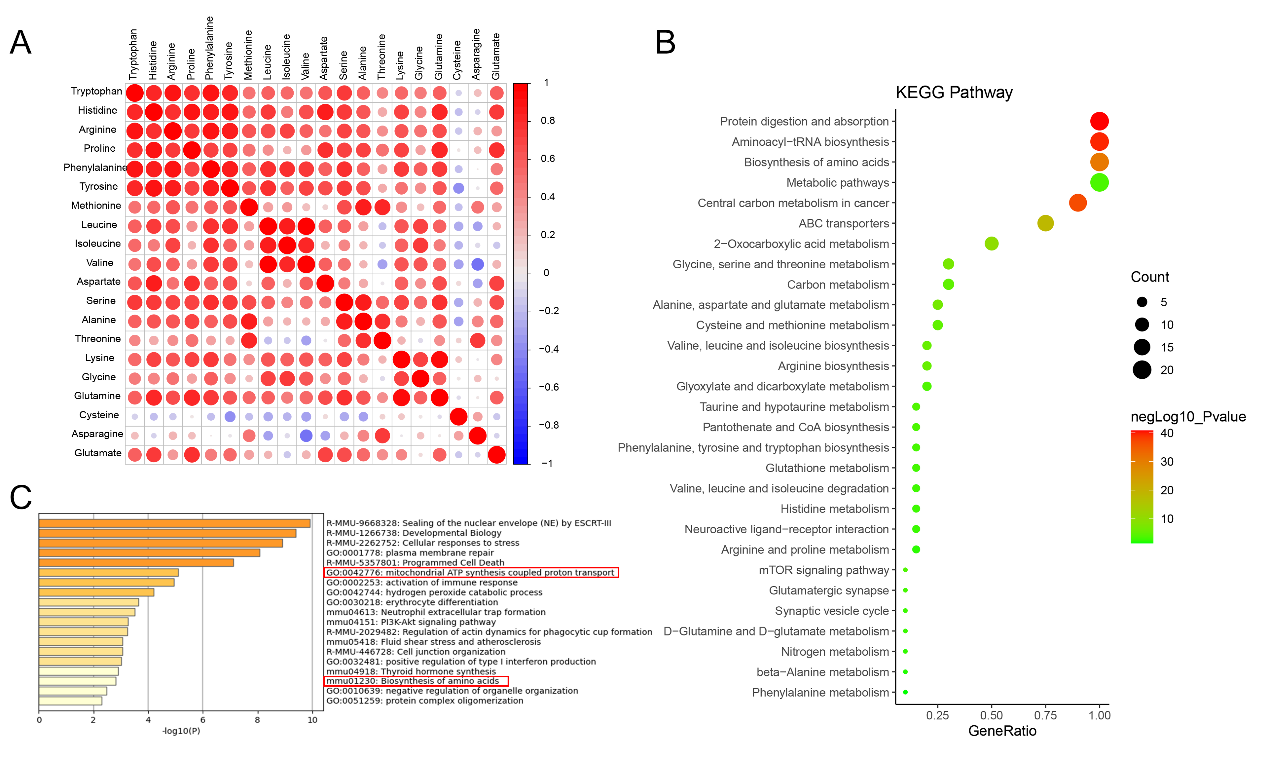
**

**Figure S2.** (A)The matrix heat map of correlation coefficient. The matrix showed the correlation between significantly differential metabolites. The Pearson correlation coefficient R was between - 1 and + 1. The correlation coefficient R between metabolites was displayed in color and circle. Red represented R>0 and indicated positive correlation; blue represented R<0 and indicated negative correlation. The larger the circle and the darker the color, the stronger the correlation. (B) The relevant pathway analysis performed by KEGG showed that the significantly differential metabolites were involved in numerous pathway participating in amino acid metabolism. (C) The enriched ontology clusters analysis of QRICH2 interacting proteins identified by Co-IP and mass spectrum indicated that QRICH2 was involved in regulating amino acid metabolism.

**
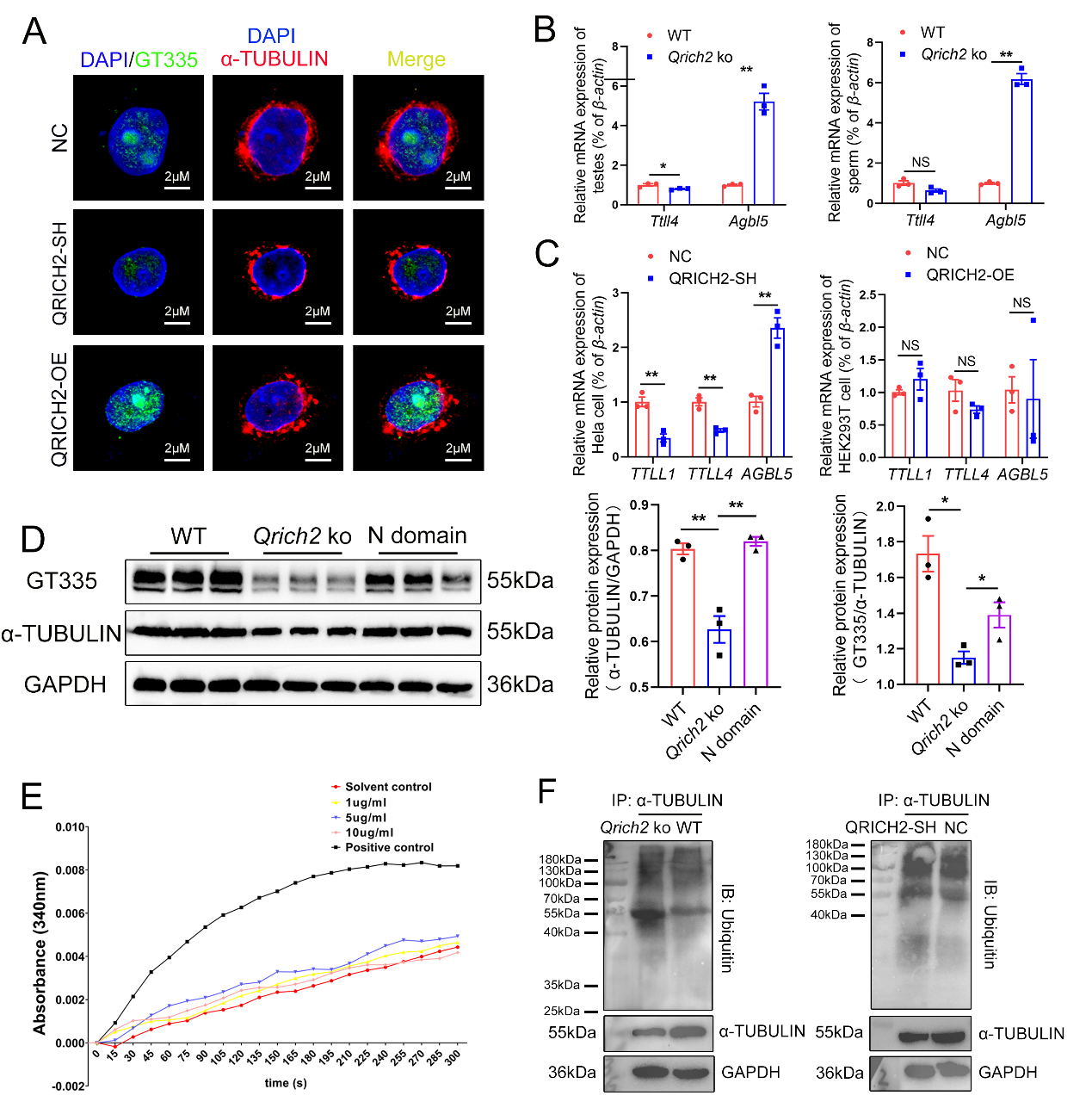
**

**Figure S3.** NC, normal control; QRICH2-SH, QRICH2 knock-down; QRICH2-OE, QRICH2 overexpression. WT, wild type; *Qrich2* KO, *Qrich2* knockout. (A) Immunofluorescence showed that knock-down of QRICH2 in Hela cells reduced the tubulin glutamylation levels, while the overexpression of QRICH2 increased the tubulin glutamylation levels. Besides, knock-down or overexpression of QRICH2 seemed to change the homogeneous distribution status of α-TUBULIN. DAPI, blue; α-TUBULIN, red; GT335, green; scale bars, 2 µm. (B) *Qrich2* KO caused disordered expression of genes involved in regulating glutamylation levels of tubulin in testes and sperm of mouse (N=3, Student’s *t* test, **P* < 0.05, ***P* < 0.01, NS, *P* > 0.05, error bars, s.e.m. (C) Knock-down of QRICH2 in hela cells induced disordered expression of genes involved in regulating glutamylation levels of tubulin, while overexpression of QRICH2 showed no significant effect on expression of these genes. N=3, Student’s *t* test, NS, *P* > 0.05, ***P* < 0.01, error bars, s.e.m. (D) Addition of the QRICH2 N-terminal purified protein *in vitro* partially restored the reduced α-TUBULIN expression and tubulin glutamylation levels induced by *Qrich2* KO. The grayscale analysis of the protein bands was shown in the right panel. N=3, Student’s *t* test, **P* < 0.05, ***P* < 0.01, error bars, s.e.m. N domain, QRICH2 N-terminal purified protein, 5µg/ml. (E) Tubulin polymerization experiment indicated that the QRICH2 N-terminal purified protein promoted the polymerization of α-TUBULIN. Solvent control represents the blank control; 10 µm paclitaxel acts as the positive control; N-terminal purified protein of 1 µg/ml, 5 µg/ml and 10 µg/ml act as the experimental group. (F) The ubiquitination levels of α-TUBULIN were increased in testes of *Qrich2* KO mice and cells of QRICH2 knock-down*.*

**
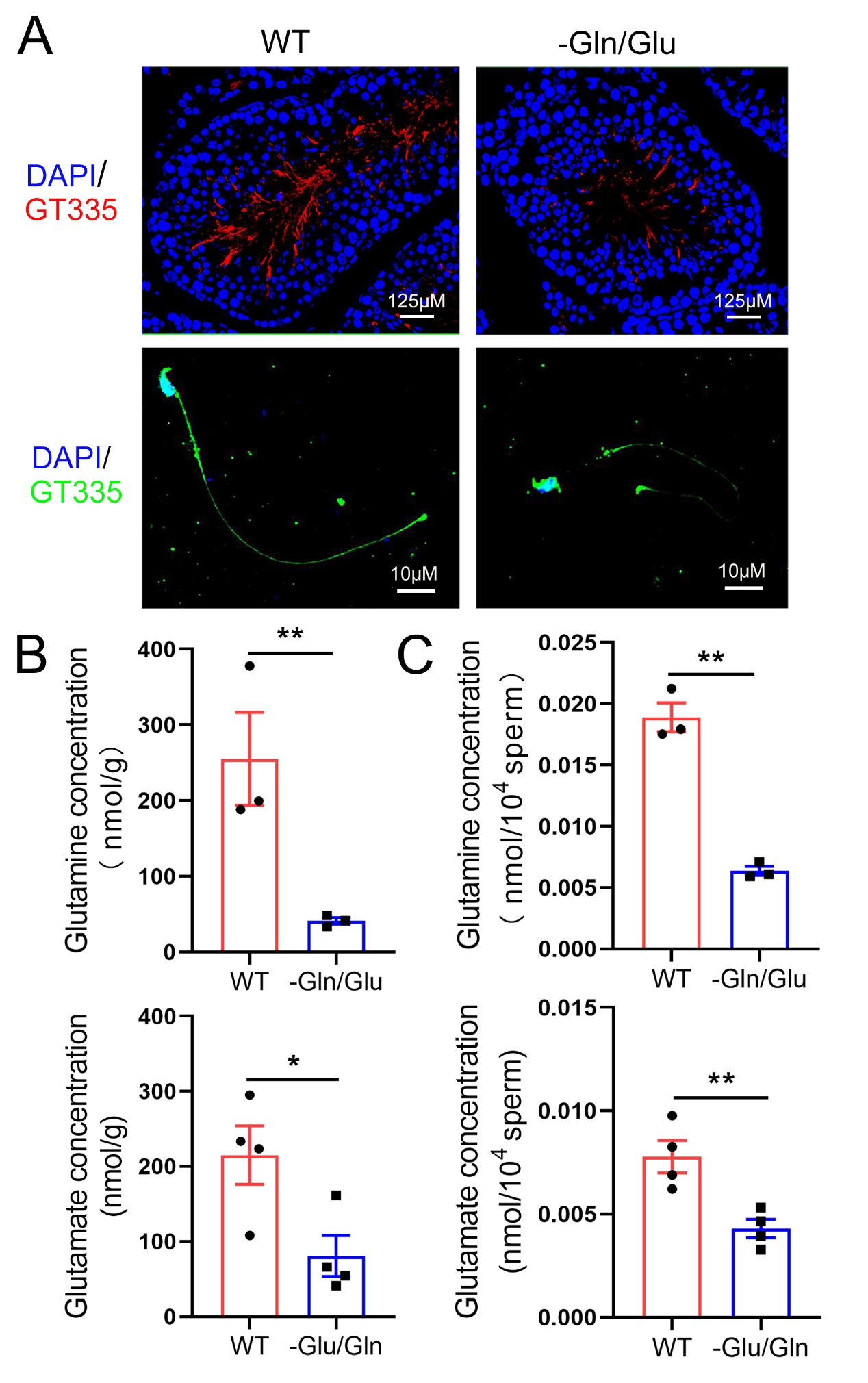
**

**Figure S4.** WT, wild type; -Gln/Glu, mice with absent Gln/Glu in diets. (A) The glutamylation levels of tubulin were decreased in testes and sperm of mice with absent Gln/Glu in diets. DAPI, blue; Upper panel, GT335, red, scale bars, 125µm; Bottom panel, GT335, green, scale bars 10µm. (B-C) The Gln and Glu concentrations were reduced in testes (B) and sperm (C) of mice with absent Gln/Glu in diets. N=3 for Gln and N=4 for Glu, Student’s *t* test, **P* < 0.05, ***P* < 0.01, error bars, s.e.m.

**
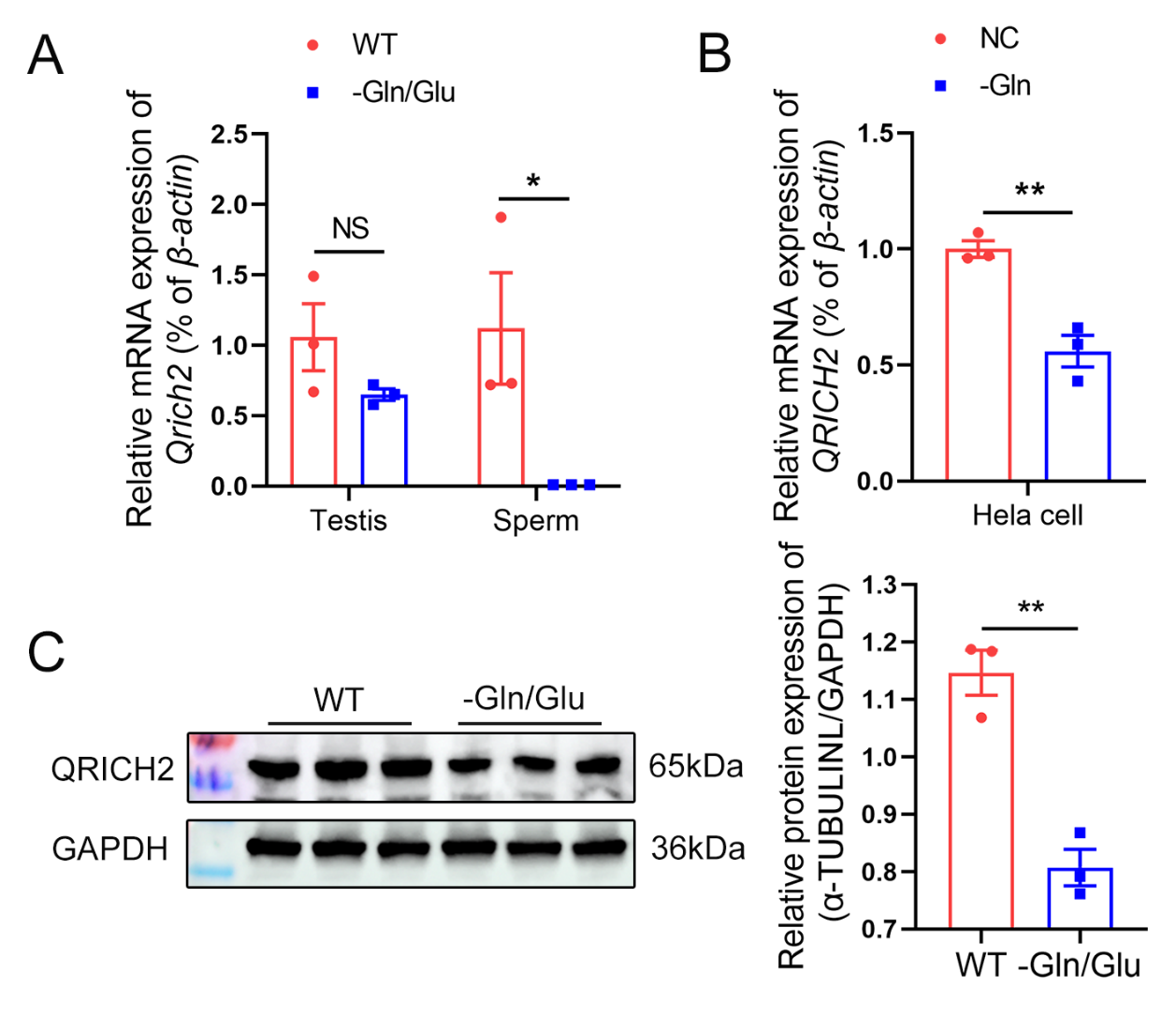
**

**Figure S5.** WT, wild type; -Gln/Glu, mice with absent Gln/Glu in diets. NC, normal control; -Gln, absence of Gln in cell culture medium. (A-C) The expression of QRICH2 was decreased in sperm (A) and testes (C) of mice with absent Gln/Glu in diets and in Hela cells (B) with absence Gln in culture medium. N=3, Student’s *t* test, **P* < 0.05, ***P* < 0.01, NS, *P* > 0.05, error bars, s.e.m.

**
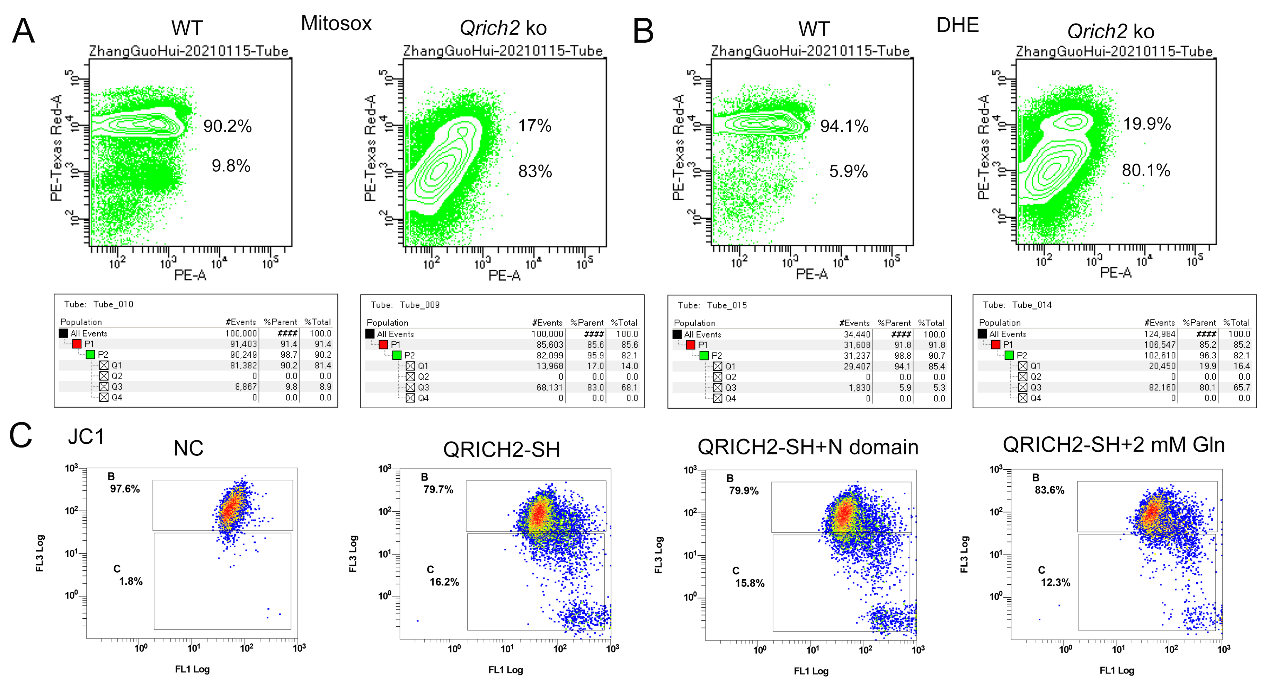
**

**Figure S6.** WT, wild type; *Qrich2* KO, *Qrich2* knockout*.* NC, normal control; QRICH2-SH, QRICH2 knock-down; N domain, QRICH2 N-terminal purified protein, 5µg/ml. (A-B) The levels of mitochondrial specific reactive oxygen species were significantly decreased in sperm of *Qrich2* KO mice. The ordinate represented the fluorescence intensity. N=3, 5000-10000 sperm were analyzed for each experiment. (C) 2 mM Gln partially rescued the QRICH2 knock-down induced decrease of mitochondrial membrane potential, whereas N-terminal purified protein of QRICH2 had no significant recovery effect. The box in the upper panel displayed the sperm with high mitochondrial membrane potential; the box in the bottom panel displayed the sperm with low mitochondrial membrane potential. N=3, 5000-10000 sperm were analyzed for each experiment.


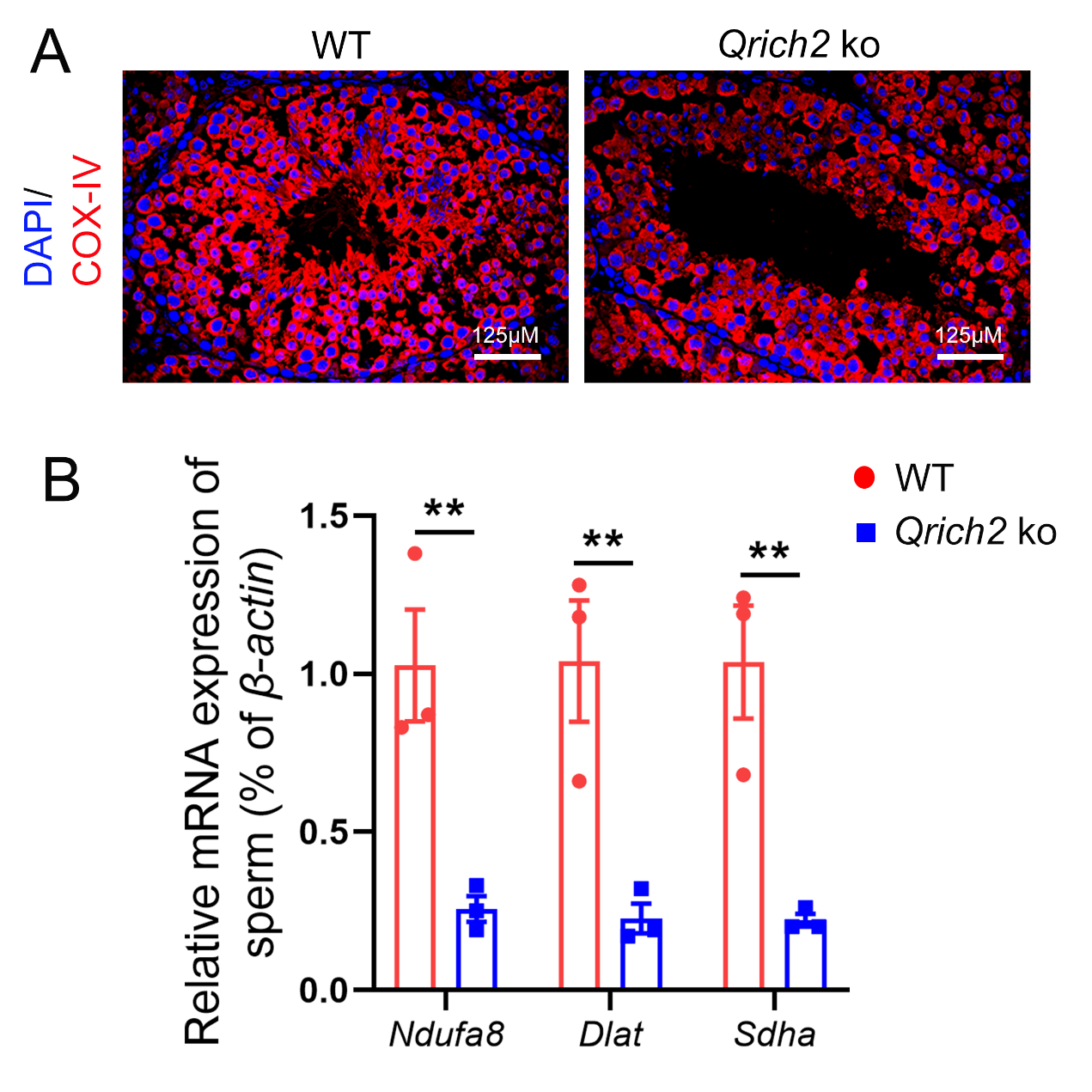


**Figure S7. The mitochondria function of sperm was decreased in *Qrich2* KO mice.** WT, wild type; *Qrich2* KO, *Qrich2* knockout; NC, normal control; QRICH2-SH, QRICH2 knock-down. (A) The expression of COX-IV was decreased in testes of *Qrich2* KO mice. DAPI, blue; COX-IV, red; scale bars, 125 µm. (B) The expression of genes related to mitochondrial oxidative phosphorylation was decreased in sperm of *Qrich2* KO mice. N=3, Student’s t test, ***P* < 0.01, error bars, s.e.m.


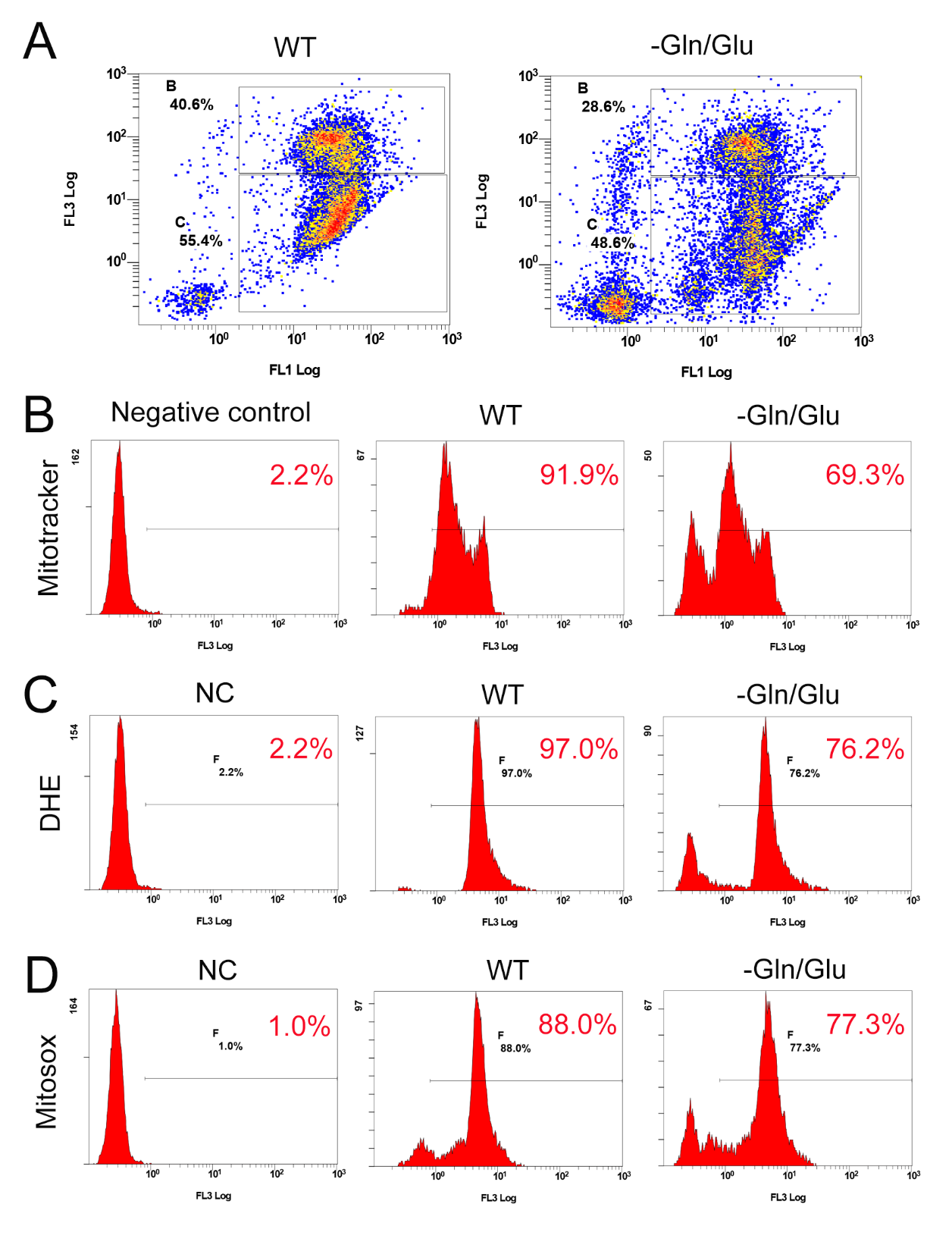


**Figure S8. The sperm mitochondria function was decreased in mice with absent Gln/Glu in diets.** WT, wild type; -Gln/Glu, mice with absent Gln/Glu in diets; (A) JC1 staining indicated the significantly decreased mitochondrial membrane potential in sperm of mice with absent Gln/Glu in diets. The box in the upper panel displayed the cells with high mitochondrial membrane potential; the box in the bottom panel displayed the cells with low mitochondrial membrane potential; the box in the lower left corner represented undetermined mitochondrial membrane potential. N=3, 5000-10000 sperm were analyzed for each experiment. (B) Mitotracker staining showed that the mitochondrial activity was decreased in sperm of mice with absent Gln/Glu in diets. The abscissa represented the fluorescence intensity and the ordinate represented the sperm quantity. N=3, 5000-10000 sperm were analyzed for each experiment. (C-D) Absence of Gln/Glu in diets induced decreased levels of mitochondrial specific reactive oxygen species in sperm of mice. The abscissa represented the fluorescence intensity and the ordinate represented the sperm quantity. N=3, 5000-10000 sperm were analyzed for each experiment.


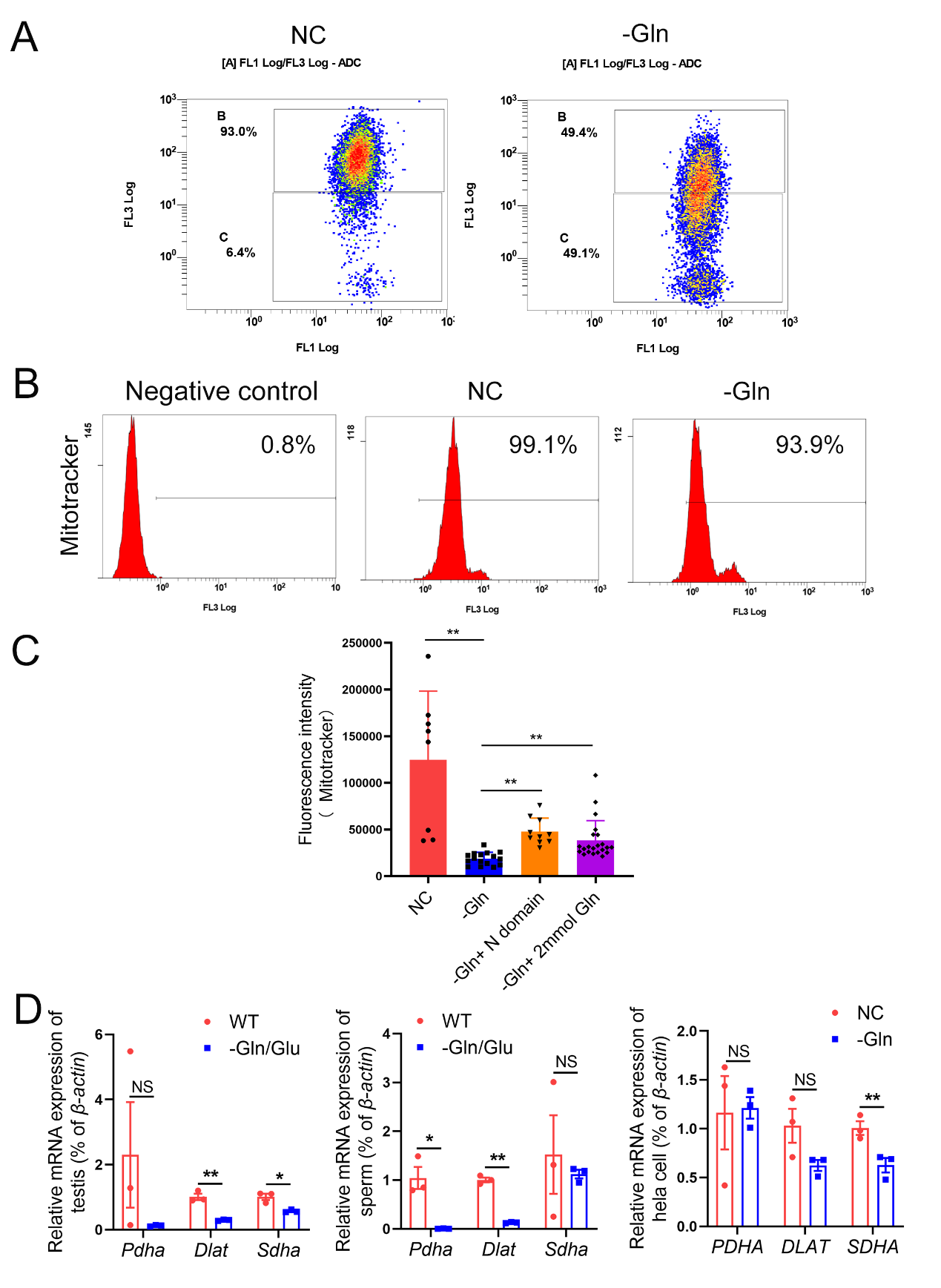


**Figure S9. The sperm mitochondria function was decreased in mice with absent Gln/Glu in diets.** WT, wild type; -Gln/Glu, mice with absent Gln/Glu in diets; NC, normal control; -Gln, absence of Gln in cell culture medium. N domain, N-terminal purified protein of QRICH2. (A) JC1 staining indicated the significantly decreased mitochondrial membrane potential in cells with absent Gln in culture medium. The box in the upper panel displayed the cells with high mitochondrial membrane potential; the box in the bottom panel displayed the cells with low mitochondrial membrane potential; the box in the lower left corner represented undetermined mitochondrial membrane potential. N=3, 5000-10000 cells were analyzed for each experiment. (B) Mitotracker staining showed that the mitochondrial activity was decreased in cells with absent Gln in culture medium. The abscissa represented the fluorescence intensity and the ordinate represented the sperm quantity. N=3, 5000-10000 cells were analyzed for each experiment. (C) Single cell metabolism analyzer showed that incubation with the N-terminal purified protein of QRICH2 (5µg/ml) or 2 mM Gln for 2 hours *in vitro* partially rescued the reduced mitochondrial function caused by absence of Gln in culture medium. The number of cells analyzed in each group was 8, 16, 10 and 22. (D) The expression of genes related to mitochondrial oxidative phosphorylation was decreased in testes (left panel) and sperm (middle panel) of mice with absent Gln/Glu in diets and in cells with absent Gln in culture medium (right panel). N=3, Student’s t test, **P* < 0.05, ***P* < 0.01, NS, *P* > 0.05, error bars, s.e.m.

**
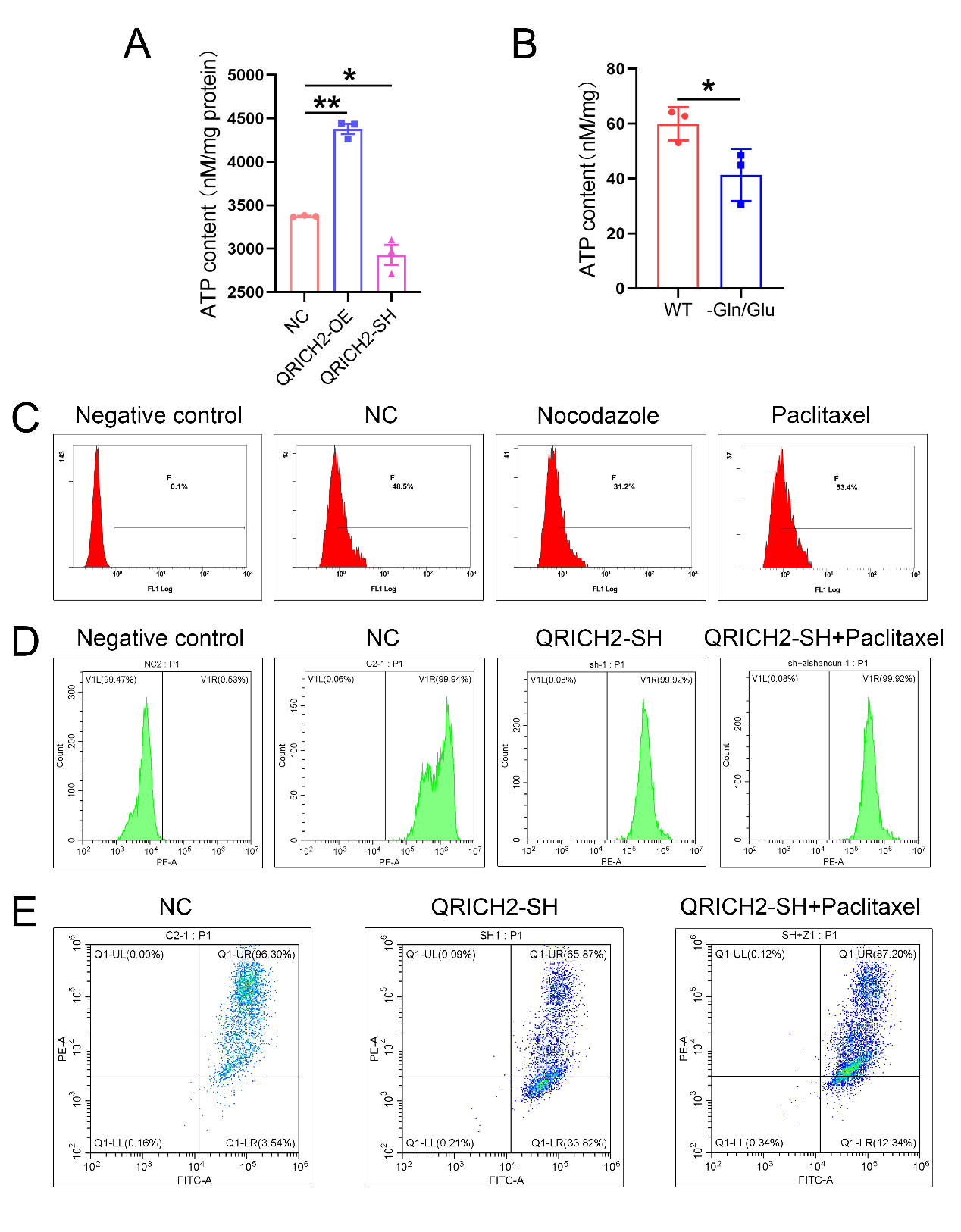
**

**Figure S10.** (A) Over expression of QRICH2 increased the ATP production whereas knockdown of QRICH2 reduced the ATP production. (B) Deficiency of Gln/Glu in diets reduced the ATP production in testes compared with wild type mice. (C) Nocodazole reduced the staining of Mitotracker whereas paclitaxel increased the staining. Paclitaxel partially rescued the reduced mitochondrial function induced by

QRICH2 knockdown including the reduced staining of Mitotracker (D) and the reduced mitochondrial membrane potential (E). QRICH2-OE, QRICH2 over expression; QRICH2-SH, QRICH2 knockdown; -Gln/Glu, deficiency of Gln/Glu in diets (N=3, Student’s t test, *P < 0.05, **P < 0.01, error bars, s.e.m.)

| Ingredients | Control diets | | Gln/Glu absence diets | |
| --- | --- | --- | --- | --- |
|  | gm | kcal | gm | kcal |
| cornstarch | 550.5 | 2202 | 585.5 | 2342 |
| Maltodextrin | 125 | 500 | 125 | 500 |
| cellulose | 50 | 0 | 50 | 0 |
| Corn oil | 50 | 450 | 50 | 450 |
| L-arginine | 10 | 40 | 40 | 40 |
| L- histidine -HCI-H2O | 6 | 24 | 6 | 24 |
| L- isoleucine | 8 | 32 | 8 | 32 |
| L- leucine | 12 | 48 | 12 | 48 |
| L- lysine -HCI | 14 | 56 | 14 | 56 |
| L- methionine | 6 | 24 | 6 | 24 |
| L- phenylalanine | 8 | 32 | 8 | 32 |
| L- threonine | 8 | 32 | 8 | 32 |
| L- tryptophan | 2 | 8 | 2 | 8 |
| L- valine | 8 | 32 | 8 | 32 |
| L- alanine | 10 | 40 | 10 | 40 |
| L- asparagine -H2O | 5 | 20 | 5 | 20 |
| L- aspartate | 10 | 40 | 10 | 40 |
| L- cystine | 4 | 16 | 4 | 16 |
| L- glutamate | 30 | 120 | 0 | 0 |
| L- glutamine | 5 | 20 | 0 | 0 |
| Glycine | 10 | 40 | 10 | 40 |
| L- proline | 5 | 20 | 5 | 20 |
| L- serine | 5 | 20 | 5 | 20 |
| L- tyrosine | 4 | 16 | 4 | 16 |
| Mixed minerals | 35 | 0 | 35 | 0 |
| Sodium bicarbonate | 7.5 | 0 | 7.5 | 0 |
| Vitamin mixture | 10 | 40 | 10 | 40 |
| Choline bitartrate | 2 | 0 | 2 | 0 |
| Total | 1000.05 | 3872 | 1000 | 3872 |
|  | gm % | kcal % | gm % | kcal % |
| Protein | 17 | 17.6 | 13.5 | 13.9 |
| Carbohydrate | 68.5 | 70.8 | 72.1 | 74.4 |
| Fat | 5 | 11.6 | 5 | 11.6 |

**Table S1. Ingredients of control and Gln/Glu absence diets**

**Table S2. The qPCR primers for mice**

| Primer sequences (5’-3’)  Mouse | Annealing (℃) |
| --- | --- |
| *Qrich2*-F: GTCCAGGGTGACTGTGAGAAG | 60 |
| *Qrich2*-R: CATCTTGGCCACCAGTTCCTG | 60 |
| *Glul*-F: GAACTTTGATGGCTCTAGTACC | 60 |
| *Glul*-R: GAGTATATTCCTGCTCCATTCC | 60 |
| *Gls1*-F: GACAACGTCAGATGGTGTCATG | 60 |
| *Gls1*-R: CCCAGGTCATTAACAGCAATTG | 60 |
| *Gls2*-F: CTGACTCAGGCATTCCGAAAG | 60 |
| *Gls2*-R: GTAGTCAGTGCCTAAGGTGCTC | 60 |
| *Ttll4*-F: TTCGGGTTTTGGTGGAGATGG | 60 |
| *Ttll4*-R: CGTGGCTGCTCAAAAAACCG | 60 |
| *Abgl5*-F: GACTGCCAGGATCTGCTGAG | 60 |
| *Abgl5*-R: GCTAGATGGAGTCTCTCCAGG | 60 |
| *Ndufa8*-F: GGCTCAGTGCGATAAAACCAAT | 60 |
| *Ndufa8*-R: CCAGTACTCTGTGAAAGGCTCC | 60 |
| *Dlat*-F: GTGAGGGTGATCTAATTGCAG | 60 |
| *Dlat*-R: CATATGAGTAGGATAGGAGCTAC | 60 |
| *Sdha*-F: CGCTGGTGTGGATGTCACTAAG | 60 |
| *Sdha*-R: GGCATACAGACCAGGCACAATC | 60 |
| *Gapdh*-F: CATCACTGCCACCCAGAAGACTG | 60 |
| *Gapdh*-R: ATGCCAGTGAGCTTCCCGTTCAG | 60 |
| *β-actin*-F: CATTGCTGACAGGATGCAGAAGG | 60 |
| *β-actin*-R: TGCTGGAAGGTGGACAGTGAGG | 60 |

**Table S3. The qPCR primers for humans**

| Primer sequences (5’-3’) | Annealing (℃) |
| --- | --- |
| Human |  |
| *QRICH2*-F: CAAGCAACCAAGTATTGAGCAG | 60 |
| *QRICH2*-R: CTCCTGTGACGATCTGAGTCTG | 60 |
| *GLUL*-F: CAATCGAAGGCCTGCAGAGA | 60 |
| *GLUL* -R: TACAAGCAGGCCCGGTAATG | 60 |
| *GLS1*-F: ACTGAGCCCTGAAGCAGTTC | 60 |
| *GLS1*-R: AAGGAATGCCTTTGATCACCAC | 60 |
| *GLS2*-F: AGCAATGCCACGGAAGGTAA | 60 |
| *GLS2*-R: CTGGAATCTGAAGCAAACACCC | 60 |
| *TTLL4*-F: ATGAGTTTTCTCGCCGTGGT | 60 |
| *TTLL4*-R: GGAAAGGCCAGCTTGAGTCT | 60 |
| *ABGL5*-F: TCTTGTTTCCTGGCAGAGCG | 60 |
| *ABGL5*-R: CTCCACGTGGGCTAGATTCC | 60 |
| *TTLL1*-F: TGAGTGTGCAAACCATCCGA | 60 |
| *TTLL1*-R: AACAGGTTGTAGTCAGCGGG | 60 |
| *GAPDH*-F: CCCATGGCAAATTCCATGGC | 60 |
| *GAPDH* -R: GCTGATGATCTTGAGGCTGT | 60 |
| *β-ACTIN*-F: GGATTCCTATGTGGGCGACG | 60 |
| *β- ACTIN* -R: GTACATGGCTGGGGTGTTGA | 60 |
| *PDHA*-F: CTGGTAGCATCCCGTAATTT | 60 |
| *PDHA*-R: ATGGTCTGTGGGGTTGATGC | 60 |
| *DLAT*-F: AACAGCGTGACTACAGGGTA | 60 |
| *DLAT*-R: CAACCTCTGCAATTAGGTCA | 60 |
| *SDHA*-F: CACTGTTGATGGGAACAAGA | 60 |
| *SDHA*-R: TGTAGTGGATGGCATCCTGG | 60 |

**Table S4. The primary and secondary antibodies involved in our study**

| Antibodies | Name | Brand | Catalog number | Dilution ratio |
| --- | --- | --- | --- | --- |
| Primary antibodies | α-TUBULIN | Abclonal | AC007 | IF (1:200) WB (1:5000)  IF (1:200) WB (1:5000) |
|  |  |  | AC012 |  |
|  | QRICH2 | [Sigma-Aldrich](https://www.so.com/link?m=bAx2KaDvN9LjC7cHWfe6VAvDnHaxM56zE%2Fw6OHtVkH1DCJlp0k9a0%2FdtPbUL4mJQVoEMaKFOXbgFd89aKOiDxhYfHAe8trhYT53nivJqtJQUU8L0Z) | [HPA052219](https://www.sigmaaldrich.cn/CN/zh/product/sigma/hpa052219) | IF (1:100)  WB (1:1000) |
|  | [Anti-polyglutamylation modification](https://adipogen.com/ag-20b-0020-anti-polyglutamylation-modification-mab-gt335.html) | Adipoge | GT335 | IF (1:200) WB (1:2000) |
|  | GLUL | Abclonal | A5437 | IF (1:200) WB (1:2000) |
|  | COX-IV | ZEN-BIOSCIENCE | 250135 | IF (1:100) WB (1:1000) |
|  | TFAM | Proteintech | 22586-1-AP | IF (1:200) |
|  | Ubiquitin | Proteintech | 10201-2-AP | WB (1:1000) |
|  | GAPDH | Abclonal | A19056 | WB (1:5000) |
| Secondary antibodies | Alexa Fluor 488 | Thermo Fisher | A21206 | IF (1:1000) |
|  | Alexa Fluor 594 | Thermo Fisher | A11005 | IF (1:1000) |
|  | Goat anti mouse secondary antibody | Thermo Fisher | G-21040 | WB (1:5000) |
|  | Goat anti rabbit secondary antibody | Thermo Fisher | 31460 | WB (1:5000) |

**Table S5. The sequences selected for constructing the phylogenetic tree of QRICH2**

| Type | Species | Chromosome | Start_site | End_site | Length |
| --- | --- | --- | --- | --- | --- |
| Fishes | *Acipenser_ruthenus | NC_048340.1 | 10724740 | 10766350 | 41610 |
| Reptiles | Anolis_carolinensis | NC_014777.1 | 112447189 | 112482405 | 35216 |
|  | Chrysemys_picta | NW_007359887.1 | 11144951 | 11199420 | 54469 |
|  | *Thamnophis_elegans | NC_045542.1 | 55432553 | 55501471 | 68918 |
| Birds | Anas_platyrhynchos | NC_040064.1 | 3282136 | 3286343 | 4207 |
|  | Taeniopygia_guttata | NC_045017.1 | 10967376 | 10973915 | 6539 |
|  | Taeniopygia_guttata | NC_045017.1 | 7614847 | 7619661 | 4814 |
|  | Taeniopygia_guttata | NC_045027.1 | 67078512 | 67084510 | 5998 |
|  | *Melopsittacus_undulatus | NC_047530.1 | 20718452 | 20724588 | 6136 |
|  | *Melopsittacus_undulatus | NC_047530.1 | 57730856 | 57737224 | 6368 |
|  | *Melopsittacus_undulatus | NC_047530.1 | 59419411 | 59425834 | 6423 |
|  | *Melopsittacus_undulatus | NC_047531.1 | 74967647 | 74973978 | 6331 |
|  | *Melopsittacus_undulatus | NC_047531.1 | 74982630 | 74989026 | 6396 |
|  | *Melopsittacus_undulatus | NC_047531.1 | 75033577 | 75039974 | 6397 |
|  | *Melopsittacus_undulatus | NC_047531.1 | 75048553 | 75054947 | 6394 |
|  | *Melopsittacus_undulatus | NW_022994137.1 | 28646 | 34964 | 6318 |
| Mammals | *Ailuropoda_melanoleuca | NC_048230.1 | 40536521 | 40570398 | 33877 |
|  | *Homo_sapiens | 17 | 76275814 | 76307998 | 32184 |
|  | *Mus_musculus | 11 | 116441358 | 116459117 | 17759 |
| Amphibians | Rhacophorus_reinwardtii | Chr12 | 747374 | 802774 | 55400 |
|  | *Xenopus_laevis | NC_030686.2 | 45171580 | 45185213 | 13633 |
| The “*” marked sequence is a reverse complementary sequence | | | | | |
